# The crotonylation reader DPF2 promotes the development and progression of colon adenocarcinoma through cell-type-specific immune regulation and metabolic reprogramming

**DOI:** 10.3389/fphar.2026.1835967

**Published:** 2026-05-18

**Authors:** Jingzhi Wang, Jingyi Zhou, Lin Liu

**Affiliations:** 1 Department of Oncology, Zhongda Hospital, School of Medicine, Southeast University, Nanjing, Jiangsu, China; 2 Department of Radiotherapy Oncology, The First People’s Hospital of Yancheng, Yancheng No.1 People’s Hospital, Affiliated Hospital of Medical School, Nanjing University, Yancheng, Jiangsu, China

**Keywords:** colon adenocarcinoma, crotonylation, DPF2, metabolic reprogramming, tumor immune microenvironment

## Abstract

**Background:**

The crotonylation reader DPF2 has been implicated in tumor progression, but its role in colon adenocarcinoma (COAD), especially in cell-type-specific immune regulation and metabolic reprogramming, remains unclear.

**Methods:**

We conducted an integrative multi-omics study combining genetic association analysis for COAD, public transcriptomic validation data, CRC-context single-cell and spatial transcriptomic datasets used as supportive expression evidence, circulating metabolite profiling, and clinical immunohistochemistry. Two-sample Mendelian randomization was used to evaluate the association between genetically predicted expression of crotonylation-related genes and COAD risk, followed by validation and mechanistic analyses centered on DPF2.

**Results:**

Among 16 crotonylation-related candidate genes with available cis-eQTL evidence, only DPF2 showed a significant positive association with COAD risk. DPF2 was consistently upregulated in GEO datasets, TCGA-COAD, and clinical samples, and high DPF2 expression was associated with advanced disease and poor overall survival. Single-cell MR identified the strongest risk-associated signal in TCL1A^+^ FCER2^+^ B cells, with a suggestive association in CD8^+^ S100B^+^ T cells. Supportive analyses using public CRC-context single-cell and spatial transcriptomic datasets further indicated heterogeneous immune-cell expression and tumor-region enrichment of DPF2. In addition, DPF2 was significantly associated with 11 COAD-related circulating metabolites, and mediation analyses suggested partial mediation through Ximenoylcarnitine (C26:1), 16α-hydroxy DHEA 3-sulfate, and Andro steroid monosulfate C19H28O6S (1).

**Conclusion:**

DPF2 is a crotonylation reader associated with COAD risk and progression. Its effects may involve cell-type-specific immune regulation and partial mediation through metabolic reprogramming. While the primary causal inference of this study focused on COAD, complementary single-cell and spatial transcriptomic findings from broader CRC datasets provided supportive expression-level evidence consistent with these associations.

## 1 Introduction

Colorectal cancer (CRC) is one of the most common and deadliest malignancies of the digestive system worldwide, and colon adenocarcinoma (COAD) is one of its most prevalent histological subtypes. According to global cancer statistics released by the International Agency for Research on Cancer (IARC), CRC ranks third in incidence and second in cancer-related mortality worldwide, second only to lung cancer (Bray et al., 2024; Siegel et al., 2022). Although substantial advances have been made in the early diagnosis, surgical management, chemotherapy, and immunotherapy of colon cancer in recent years, leading to improved survival outcomes in patients with early-stage disease, the 5-year survival rate of patients with advanced, especially metastatic, disease remains only approximately 16%, with no fundamental breakthrough having been achieved over the long term (Siegel et al., 2024). Epidemiological models further predict that, driven by population aging and lifestyle changes, the global disease burden of CRC will continue to rise over the coming decades (Morgan et al., 2023). This clinical challenge largely stems from the marked heterogeneity of the molecular mechanisms underlying CRC, the complexity of the immune regulatory network within the tumor microenvironment (TME), the limited availability of actionable therapeutic targets and predictive biomarkers, and the persistent problem of antitumor drug resistance (Shin et al., 2023; Li et al., 2024; Ferkel et al., 2025). Therefore, a deeper understanding of the molecular pathological mechanisms underlying CRC, particularly COAD, and the identification of novel molecular diagnostic biomarkers and therapeutic targets remain urgent priorities in both clinical and translational research.

In recent years, accumulating evidence has shown that epigenetic modifications and metabolic reprogramming constitute key hubs linking tumor genotype to phenotypic heterogeneity (Xu et al., 2023). Among these mechanisms, post-translational modifications (PTMs), through reversible chemical modifications of lysine residues on proteins, play critical roles in regulating gene expression, protein activity, subcellular localization, and cellular signaling pathways (Wang and Cole, 2020). Among the various types of PTMs, crotonylation (Kcr), a novel lysine acylation modification tightly coupled to cellular metabolic status, has attracted increasing attention in recent years (Wan et al., 2019; Han et al., 2025). Unlike classical acetylation, the substrate supply for Kcr directly depends on intracellular crotonyl-CoA levels, which are substantially influenced by fatty acid catabolism, lysine metabolism, and tumor-associated metabolic reprogramming (Yuan et al., 2023). Previous studies have demonstrated that alterations in crotonyl-CoA levels can directly regulate histone Kcr modification, thereby activating specific transcriptional programs involved in tumorigenesis, DNA damage responses, and the regulation of immune-related genes (Yuan et al., 2023; Zhao et al., 2022). These findings suggest that Kcr may serve as an important molecular bridge linking tumor metabolic states to immune phenotypes, and that a deeper understanding of the regulatory mechanisms of crotonylation-related factors in tumor progression may provide novel targeted strategies for cancer prevention and treatment.

The biological functions of Kcr depend on a specific “writer–eraser–reader” regulatory system (Jiang et al., 2021). Among these factors, DPF2 (Double PHD Finger Protein 2) has been identified as a key crotonylation “reader” that selectively binds crotonylated sites on histone H3 through its tandem PHD domains, thereby remodeling chromatin conformation and regulating downstream transcriptional programs (Xiong et al., 2016). Previous studies have shown that DPF2 is aberrantly expressed in multiple malignancies and significantly affects the proliferative, invasive, and metastatic capacities of tumor cells. For example, in hepatocellular carcinoma, high DPF2 expression has been reported to be significantly associated with tumor progression and poor patient survival (Yang et al., 2024a). In addition, preliminary evidence suggests that DPF2 may promote malignant progression by regulating multiple key signaling pathways involved in cell-cycle progression, DNA damage repair, and apoptosis (Zhai et al., 2024). Moreover, DPF2 has been reported to participate in the transcriptional regulation of genes related to inflammatory responses and oxidative stress, implying a potential role in tumor immune regulation (Mas et al., 2023). However, whether DPF2-mediated crotonylation regulation causally drives COAD development at the genetic level, and whether its effects are restricted to specific cell types, remains to be systematically elucidated.

Immune cells play pivotal roles in shaping the TME and driving tumor progression, and their composition and functional states are critical determinants of CRC progression and therapeutic response (Binnewies et al., 2018). Increasing evidence indicates that tumor-associated immune cells, including T cells, B cells, and macrophages, regulate tumor growth, invasion, metastasis, and immune evasion through interactions with tumor cells and other stromal components (Zhang et al., 2020). With the development of single-cell transcriptomic technologies, highly heterogeneous immune cell subsets have been identified in CRC, including functionally exhausted CD8^+^ T cells, immunoregulatory B-cell subsets, and immunosuppressive myeloid populations (Pelka et al., 2021). Further single-cell and spatial transcriptomic studies have demonstrated that distinct immune cell subsets vary substantially among patients and across different tumor stages and are closely associated with immune evasion and disease progression (Chu et al., 2024; Chen et al., 2024; Wang et al., 2023). These findings suggest that key molecular events in CRC may not be driven by the average effect across all immune cells, but rather may depend on specific immune cell subsets. Therefore, dissecting relevant molecular regulatory mechanisms at single-cell resolution is of major significance for the development of precision therapeutic strategies for CRC.

In addition to PTMs, metabolic reprogramming is another hallmark of colon adenocarcinoma (COAD) development and progression (Nicolini and Ferrari, 2024). Tumor metabolic reprogramming not only provides an energetic basis for rapidly proliferating tumor cells, but also contributes to the regulation of signaling pathways and the establishment of the TME through metabolic intermediates (Pavlova et al., 2022). Tumor cells and immune cells within the microenvironment can reshape fatty acid and amino acid metabolic pathways, thereby altering the supply of short-chain acyl-CoA and related metabolic intermediates and regulating transcriptional programs through the metabolism–epigenetics axis (Sabari et al., 2017). Beyond lipid metabolism, growing evidence suggests that circulating endocrine-related metabolites, particularly steroid hormones and their derivatives, may participate in tumor progression by modulating immune cell function, nuclear receptor signaling, and chromatin states (Anderson and Acharya, 2022), although their causal roles in COAD have not yet been systematically clarified. Given the high sensitivity of crotonylation to the availability of metabolic substrates (Xie et al., 2024), it remains unclear whether crotonylation regulators influence COAD risk through specific circulating metabolites, including lipids and endocrine-related metabolites, and what proportion of this effect is mediated by distinct metabolic pathways.

Mendelian randomization (MR), a statistical approach that uses genetic variants as instrumental variables to infer causal relationships, can reduce confounding and reverse causality to some extent (Skrivankova et al., 2021; Sanderson et al., 2022). As such, MR has increasingly become an important analytical tool for investigating causal “gene–metabolite–disease” networks in cancer research (Yun et al., 2023). However, conventional MR analyses are usually based on bulk transcriptomic or tissue-level data and are therefore unable to resolve the causal effects of gene expression in specific cell types. Integrating the MR framework with single-cell transcriptomic data offers the opportunity to reveal the potential causal contributions of key molecular events to COAD risk at the level of cellular subsets (Yazar et al., 2022; Zheng et al., 2025). To date, MR studies investigating the causal relationships among crotonylation-related gene expression, metabolic pathways, and COAD remain lacking, particularly those exploring the cell-type-specific effects of gene expression on COAD risk at the single-cell level.

Accordingly, in this study, we applied a single-cell eQTL-based Mendelian randomization framework to systematically evaluate the potential impact of the key crotonylation reader DPF2 on colon adenocarcinoma risk in specific immune cell subsets, and further explored the genetic associations and mediating effects between DPF2 and lipid-as well as steroid hormone-related metabolic pathways. Given the availability of public genetic outcome data, the primary causal inference outcome of this study focused on colon adenocarcinoma (COAD), whereas publicly available single-cell and spatial transcriptomic datasets were mainly used as supportive expression evidence in the broader CRC context. Our study aimed to systematically dissect the potential role of the crotonylation–immunity–metabolism axis in COAD development from the perspectives of cell-type specificity and causal inference, while using publicly available single-cell and spatial transcriptomic datasets in the broader CRC context as supportive expression evidence, and to provide multi-omics evidence supporting DPF2 as a genetically associated candidate biomarker and putative regulatory factor in COAD.

## 2 Methods

### 2.1 Study design and reporting framework

**FIGURE 1:**
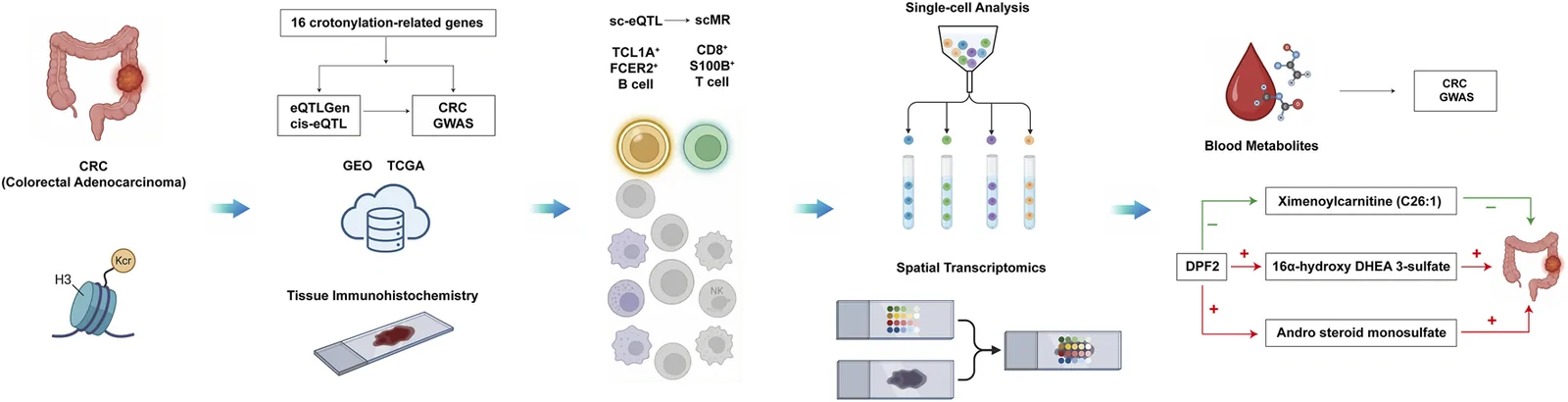
Study workflow. Schematic overview of the study design. Public transcriptomic datasets, clinical tissue immunohistochemistry, single-cell and spatial transcriptomic data, and circulating metabolite analyses were integrated to systematically evaluate the expression characteristics, cell-type specificity, and potential immune–metabolic mechanisms of DPF2 in colon adenocarcinoma (COAD). Some icons/elements in Figure 1 were created in BioRender. Wang, J. (2026) https://BioRender.com/11djg17.

This study was an integrative analysis based on publicly available summary statistics, public multi-omics datasets, and clinical tissue immunohistochemistry data (Figure 1). The study workflow included the following steps: (1) compilation of crotonylation-related candidate genes and screening for genes with available cis-eQTL evidence; (2) evaluation of the two-sample causal association between genetically predicted gene expression and colon adenocarcinoma (COAD) risk; (3) validation of the expression characteristics of key genes in tumor tissues using GEO, TCGA, and human tissue immunohistochemistry data; (4) cell-type-specific scMR analysis based on single-cell eQTL data from 14 immune cell subsets; (5) supplementary analysis of the expression distribution of key genes in the CRC immune microenvironment using public single-cell transcriptomic data; (6) assessment of the spatial localization of key genes and their relationship with malignant regions and microenvironmental components using spatial transcriptomic data; (7) systematic screening of circulating metabolites associated with COAD risk and evaluation of the genetic associations between DPF2 and these metabolites; and (8) mediation analysis to quantify the indirect effect contribution along the “DPF2–metabolite–COAD” pathway. The public database component of this study was based on published de-identified summary data or public database resources and therefore did not require additional ethical approval. The human tissue immunohistochemistry component used patient samples from Yancheng First People’s Hospital and was approved by the institutional ethics committee (2025-K(YJ)-157), with informed consent or a waiver of informed consent obtained as required.

### 2.2 Data sources

Expression quantitative trait locus (eQTL) data used for gene expression-related analyses were obtained from the eQTLGen consortium database (https://www.eqtlgen.org/cis-eqtls.html). All data used in this study were cis-expression quantitative trait loci (cis-eQTLs) derived from peripheral blood samples (Võsa et al., 2021). We additionally included single-cell eQTL data from the OneK1K cohort published by Yazar et al. in *Science* (2022). This dataset was generated by integrating single-cell transcriptomic and genotyping data from peripheral blood mononuclear cells (PBMCs) of 982 healthy individuals of Northern European ancestry, covering 14 immune cell subsets and identifying a total of 26,597 independent cis-eQTLs, which were used for cell-type-specific genetically predicted expression analyses (Yazar et al., 2022).

Public transcriptomic validation data included colon adenocarcinoma-related expression profiling datasets from GEO and TCGA, which were used to validate the expression characteristics of key genes at the transcriptomic level. Protein expression validation data were derived from tissue immunohistochemistry data from patients at Yancheng First People’s Hospital and were used to further validate the expression characteristics of key genes at the protein level.

Public single-cell RNA sequencing data were obtained from the TISCH2 database, and the GSE146771 dataset (10x Genomics platform) was selected to supplement the analysis of the cellular distribution of key genes in the CRC immune microenvironment. Spatial transcriptomic data were obtained from the Sparkle database and were used to assess the spatial localization of key gene expression and its relationship with local microenvironmental components.

Metabolite-related genetic instruments were obtained from the publicly available GWAS Catalog. The original data were derived from a GWAS including more than 8,000 individuals of European ancestry and covered 1,400 metabolite traits (1,091 blood metabolites and 309 metabolite ratios; GWAS Catalog accession numbers: GCST90199640–GCST90201019) (Chen et al., 2023).

The outcome data consisted of GWAS summary statistics for colon adenocarcinoma (COAD) risk from the FinnGen R12 database, specifically the dataset finngen_R12_C3_COLON_ADENO_EXALLC.gz, which included 4,478 cases and 378,749 controls.

### 2.3 Compilation of crotonylation-related genes and screening for available eQTL evidence

Based on previous review articles on lysine crotonylation regulators, we compiled a list of 34 crotonylation-related genes (Supplementary Table S1) (Yang et al., 2024b) and standardized the gene names. We then intersected this gene set with genes showing robust cis-eQTL signals in the eQTLGen database. Ultimately, 16 crotonylation-related candidate genes with both available genetic instrumental variables and expression regulatory evidence were identified for subsequent genetic causal inference analyses (Supplementary Figure S1; Supplementary Table S2).

### 2.4 Instrumental variable selection, LD clumping, and data harmonization

To construct robust genetic instrumental variables (IVs), we applied a unified SNP screening and harmonization pipeline for all exposure traits (gene expression or metabolites). First, QTL loci reaching the genome-wide significance threshold were preferentially selected as IVs. For gene expression- and metabolite-related analyses, the primary selection threshold was set at *P* < 5 × 10^−8^; when original studies recommended different thresholds for specific traits, the original study criteria were followed. Second, linkage disequilibrium (LD) clumping was performed using population-matched LD reference panels, with parameters of *r* ^2^ < 0.1 and a window size of 10,000 kb, to retain independent instrumental SNPs. Exposure and outcome datasets were then harmonized to align the effect alleles. Palindromic SNPs lacking allele frequency information and for which strand orientation could not be reliably determined were excluded. To reduce the risk of weak instrument bias, F-statistics were calculated, and only SNPs with F > 10 were retained. If an exposure SNP was unavailable in the outcome dataset, a high-LD proxy SNP (*r* ^2^ > 0.8) was used when available, while maintaining allele alignment consistency.

### 2.5 Genetically predicted expression of 16 crotonylation-related genes and assessment of colon adenocarcinoma risk

For the 16 screened crotonylation-related candidate genes, two-sample Mendelian randomization (MR) analysis was conducted using cis-eQTLs from the eQTLGen database as instrumental variables, genetically predicted gene expression as the exposure, and COAD GWAS data as the outcome. The primary analysis used the inverse-variance weighted (IVW) method to evaluate the association between gene expression and COAD risk. When only one eligible instrumental single-nucleotide polymorphism (SNP) was available for a given gene, the Wald ratio method was used to estimate the effect. The results were reported as effect size (β), standard error (SE), odds ratio (OR), 95% confidence interval (CI), and the number of instrumental SNPs included in the analysis (nsnp). To control for type I error due to multiple comparisons, Bonferroni correction was applied across the 16 candidate genes, with a significance threshold of 0.05/16. Nominally significant associations were also retained as suggestive signals. Genes meeting the predefined criteria were subsequently included in downstream expression validation and mechanistic analyses.

### 2.6 Validation of differential DPF2 expression in GEO cohorts and TCGA

To validate the expression changes of DPF2 in colon adenocarcinoma, we performed differential expression analyses using GEO datasets and TCGA-COAD data. The GEO component included the GSE4183, GSE8671, GSE20916, GSE32323, and GSE37364 datasets, which were used to compare DPF2 expression between normal colon tissues and colon adenoma or colon adenocarcinoma tissues. For microarray data, probes were first annotated to the gene level, and the median value of duplicate probes was used as the gene expression value. Necessary log2 transformation and normalization were then performed according to the data distribution. Prespecified groups were extracted based on sample annotations, and Wilcoxon rank-sum tests were used to compare DPF2 expression differences across groups. For the TCGA component, paired tumor-adjacent normal and tumor expression data from TCGA-COAD were used to compare DPF2 expression between tumor tissues and adjacent normal tissues, with statistical assessment performed using the paired Wilcoxon signed-rank test. These analyses were used to validate the aberrant expression characteristics of DPF2 in colon adenocarcinoma at the level of independent public cohorts.

### 2.7 Immunohistochemistry and survival analysis in human tissues

To validate the protein-level expression characteristics of DPF2 in colon adenocarcinoma, immunohistochemical staining was performed on formalin-fixed paraffin-embedded tissue samples from patients. Tissue sections were deparaffinized, rehydrated, subjected to antigen retrieval, and blocked, followed by overnight incubation at 4 °C with a primary anti-DPF2 antibody (ZEN-BIOSCIENCE, Chengdu, China; Cat. No. R30152; 1:200). This was followed by incubation with a secondary antibody, DAB visualization, and hematoxylin counterstaining. Immunohistochemical results were independently evaluated by two physicians using a semiquantitative scoring approach adapted from the immunoreactive score (IRS) principle (Fedchenko and Reifenrath, 2014). Staining intensity was scored on a scale of 0–3, and the proportion of positive cells was also scored on a scale of 0–3. The final IHC score was calculated as the product of these two scores. Based on the predefined cutoff, samples were categorized into a low-DPF2 expression group (<6) and a high-DPF2 expression group (≥6), with reference to previous semiquantitative immunohistochemical categorization strategies used in colorectal cancer research (Ling et al., 2017). To evaluate the relationship between DPF2 expression and clinicopathological features, age, sex, differentiation grade, TNM stage, lymph node metastasis, distant metastasis, recurrence, and survival status at last follow-up were compared between the two groups. Continuous variables were presented as median (interquartile range, IQR) and compared using the Mann–Whitney U test. Categorical variables were presented as frequency (percentage) and compared using the Pearson χ^2^ test or Fisher’s exact test. For patients with follow-up data, postoperative overall survival (OS) analysis was further performed according to the predefined DPF2 expression groups. OS was defined as the time from surgery to death from any cause or to the date of last follow-up for censored patients. Kaplan–Meier survival curves were plotted, and differences in OS between the high- and low-expression groups were compared using the log-rank test.

### 2.8 Risk assessment of DPF2 across immune cell subsets based on sc-eQTL data

To dissect the cell-type specificity of the risk effect of the key candidate gene, we constructed cell-type-specific expression instrumental variables based on single-cell expression quantitative trait loci (sc-eQTL) data from 14 immune cell subsets. Genetically predicted gene expression in each immune cell subset was used as the exposure, and colon adenocarcinoma GWAS data were used as the outcome in two-sample MR analysis. Only immune cell subsets with at least three instrumental single-nucleotide polymorphisms (SNPs) were included in the analysis. The primary analysis used the inverse-variance weighted (IVW) method to assess the potential causal association between gene expression and colon adenocarcinoma risk. To control for multiple testing bias arising from parallel comparisons across 14 immune cell subsets, Bonferroni correction was applied, with the significance threshold set at *P* < 0.05/14. Results not meeting the Bonferroni-corrected threshold but with *P* < 0.05 were defined as nominally significant.

### 2.9 Systematic screening of circulating metabolites associated with colon adenocarcinoma risk

To systematically characterize the genetic metabolic features associated with colon adenocarcinoma, we conducted two-sample causal effect analyses using circulating metabolite GWAS summary statistics, with each circulating metabolite treated as the exposure and colon adenocarcinoma as the outcome. Instrumental variable selection, LD clumping, F-statistic filtering, and data harmonization followed the procedures described in Section 2.4. To enhance the robustness of effect estimation and the reliability of interpretation, only metabolites with at least three eligible instrumental single-nucleotide polymorphisms (SNPs) were included in the subsequent analyses. The primary analysis used the inverse-variance weighted (IVW) method to evaluate the potential causal association between circulating metabolites and colon adenocarcinoma risk.

### 2.10 Genetic association analysis between DPF2 and COAD-related metabolites

After identifying candidate metabolites associated with COAD risk, we further performed two-sample MR analysis using genetically predicted DPF2 expression as the exposure and each relevant metabolite as the outcome to evaluate the potential genetic effect of DPF2 on COAD-related metabolites. The primary analysis used the inverse-variance weighted (IVW) method for effect estimation. For analyses including at least three instrumental SNPs, sensitivity analyses were additionally performed to assess robustness and directional consistency. As this step represented downstream analysis of candidate metabolites, *P* < 0.05 was used as the nominal significance threshold.

### 2.11 Mediation analysis: DPF2–metabolite–colon adenocarcinoma

To evaluate the potential mediating roles of metabolites in the effect of DPF2 on COAD risk, we adopted a two-step mediation analysis framework based on summary statistics. First, genetically predicted DPF2 expression was used as the exposure and the target metabolite as the outcome to estimate the effect of DPF2 on the metabolite (β1). Second, the target metabolite was used as the exposure and COAD as the outcome to estimate the effect of the metabolite on COAD risk (β2). The indirect effect was then defined as the product of β1 and β2. The total effect was derived from the primary IVW estimate of DPF2 on COAD risk, and the mediation proportion was defined as the ratio of the indirect effect to the total effect. The standard error of the indirect effect was approximated using the first-order Delta method, based on which a Z statistic was constructed. Two-sided *P* values were calculated based on the standard normal distribution, and 95% confidence intervals were estimated using the normal approximation method.

### 2.12 Sensitivity analysis and robustness assessment

To assess the robustness of the two-sample MR results, sensitivity analyses were performed for analyses including at least three instrumental single-nucleotide polymorphisms (SNPs). The weighted median, MR-Egger regression, and weighted mode methods were used as complementary approaches for effect estimation, enabling comparison of effect direction and magnitude across different methods. Cochran’s Q test was used to assess heterogeneity among instrumental SNPs, the MR-Egger intercept test was used to evaluate directional horizontal pleiotropy, and leave-one-out analysis was conducted to determine whether the results were disproportionately driven by any single SNP. If different methods yielded consistent effect directions and no substantial heterogeneity or horizontal pleiotropy was detected, the results were considered robust.

### 2.13 Supplementary single-cell transcriptomic analysis

To provide supplementary support for the genetic association results from the perspective of expression distribution, we further analyzed public CRC single-cell RNA sequencing data. Data were obtained from the TISCH2 database, and the GSE146771 dataset (10x Genomics platform) was selected for analysis. Cell type annotations were provided by the database and cross-checked using the expression of canonical marker genes. Based on the single-cell expression matrix and cell annotation results, UMAP was used to visualize the dimensionality reduction of major cell populations, and the expression distribution of DPF2 across different cell types was analyzed. In addition, the average expression level in each cell population was summarized using bar plots. Overall differences in DPF2 expression across cell types were assessed using the Kruskal–Wallis rank-sum test.

### 2.14 Spatial transcriptomic analysis

To evaluate the spatial localization of DPF2 expression and its relationship with microenvironmental components in the tissue context, we analyzed colorectal cancer spatial transcriptomic data from the Sparkle database. This database provides uniformly processed spot-level expression matrices and cell-type deconvolution results. Based on the overall expression pattern of DPF2, four representative sections—Human Intestine Cancer, ST-colon1, ST-colon3, and ST-colon4—were selected for subsequent analyses. For each spot, the dominant cell type was defined as the cell component with the highest proportion, and the average DPF2 expression was calculated across regions dominated by different cell types. To facilitate cross-section comparison, heatmaps were generated after Z-score standardization of the average expression values within each section. The spatial distributions of dominant cell types were then visualized to characterize the spatial composition of tumor, immune, and stromal components in different samples. Based on the proportion of TumorCells in the deconvolution results, all spots were uniformly classified into malignant regions (Mal; TumorCells >0) and non-malignant regions (nMal; TumorCells = 0), and these two spatial categories were visualized. The Wilcoxon rank-sum test was used to evaluate differences in DPF2 expression between Mal and nMal regions, and bar plots were used to display the mean expression and standard error of the two groups. In addition, Spearman correlation coefficients were calculated at the spot level to analyze the relationship between DPF2 expression and the proportions of different cell types, while also assessing the correlation structure among cell types.

### 2.15 Statistical software

Statistical analyses were primarily conducted using R software (version 4.5.0), including data processing, statistical analysis, and figure generation. The main R packages used in this study included TwoSampleMR, ggplot2, survival, and survminer.

## 3 Results

### 3.1 Genetic screening of crotonylation-related candidate genes identified DPF2 as a core gene associated with colon adenocarcinoma risk

To systematically evaluate the potential roles of crotonylation-related genes in the development and progression of colon adenocarcinoma (COAD), we first performed two-sample Mendelian randomization analyses on the 16 screened crotonylation-related candidate genes. Using cis-eQTLs from the eQTLGen database as exposures and COAD GWAS data as the outcome, the inverse-variance weighted (IVW) method was applied as the primary analytical approach to assess the associations between genetically predicted expression levels of each gene and COAD risk.

**FIGURE 2:**
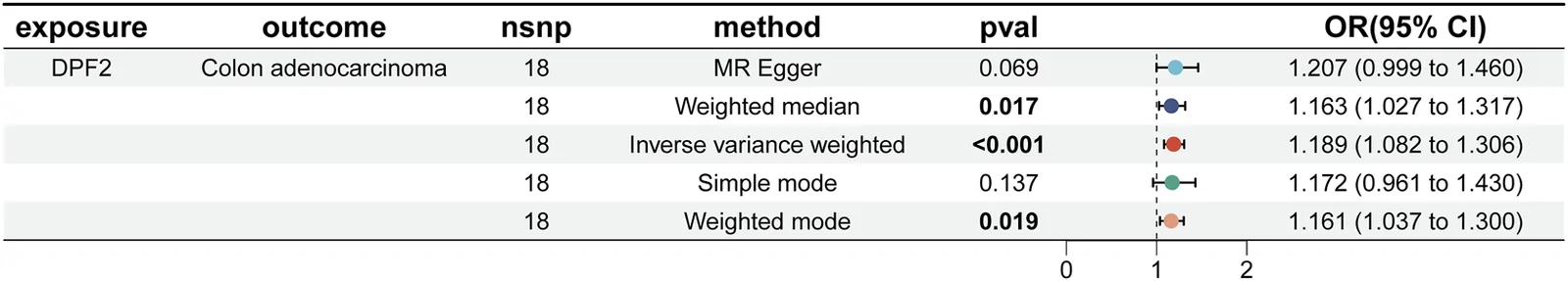
Association between genetically predicted DPF2 expression and the risk of colon adenocarcinoma. Two-sample Mendelian randomization analysis showing the association between genetically predicted DPF2 expression and COAD risk. Elevated DPF2 expression was significantly associated with an increased risk of COAD.

Among the 16 crotonylation-related candidate genes, only genetically predicted DPF2 expression showed a significant positive association with COAD risk. The IVW analysis demonstrated that elevated DPF2 expression was significantly associated with an increased risk of COAD (β = 0.173, SE = 0.048, OR = 1.189, 95% CI 1.082–1.306, *P* < 0.001), exceeding the Bonferroni-corrected threshold (0.003125) (Figure 2). Sensitivity analyses showed that the effect directions obtained from different MR methods were consistent, indicating good robustness of this association (Supplementary Tables S3,S4).

### 3.2 Multi-cohort and clinical validation confirmed high DPF2 expression in colon adenocarcinoma and indicated poor prognosis

**FIGURE 3:**
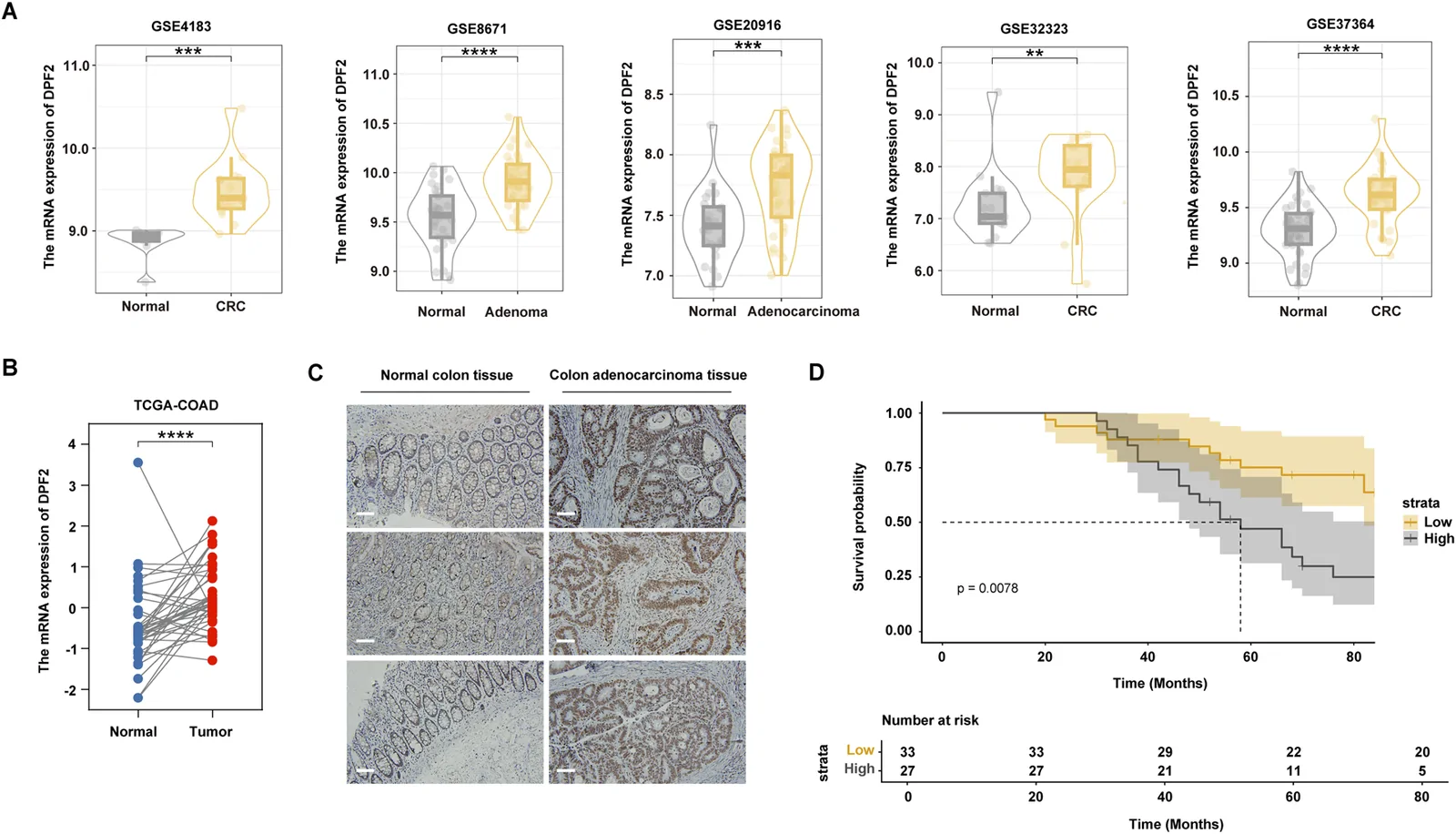
Validation of DPF2 expression and prognostic significance in colon adenocarcinoma. **(A)** Differential expression of DPF2 in normal colon tissues, adenoma tissues, and colon adenocarcinoma tissues across GEO cohorts. **(B)** Comparison of DPF2 expression between adjacent normal tissues and tumor tissues in the TCGA-COAD cohort. **(C)** Representative immunohistochemical staining images with scale bars (100 µm) showing DPF2 protein expression in normal colon tissues and colon adenocarcinoma tissues. **(D)** Kaplan–Meier overall survival analysis stratified by DPF2 expression level.

Based on the genetic association screening, we next performed multi-level validation using GEO, TCGA-COAD, and clinical samples to further verify the association between DPF2 and colon adenocarcinoma at the expression level. Results from the GEO cohorts showed an overall upregulation trend of DPF2 in diseased tissues across five independent datasets. Compared with normal colon tissues, DPF2 expression was significantly increased in CRC tissues in GSE4183, GSE32323, and GSE37364, and was also significantly elevated in adenoma tissues in GSE8671 and adenocarcinoma tissues in GSE20916 (Figure 3A). These results suggest that the aberrant upregulation of DPF2 is not restricted to fully developed carcinoma tissues, but may already emerge during the early stage of the adenoma-to-adenocarcinoma transition. Further validation in the TCGA-COAD cohort revealed that DPF2 mRNA expression in tumor tissues was significantly higher than that in adjacent normal tissues (Figure 3B), further supporting the aberrant overexpression of DPF2 in colon adenocarcinoma at the level of independent public cohorts.

To further validate DPF2 expression at the protein level, immunohistochemical staining was performed in clinical colon adenocarcinoma tissues and adjacent normal tissues. Representative immunohistochemistry images showed stronger DPF2 staining in colon adenocarcinoma tissues than in normal colon tissues (Figure 3C), indicating an upregulated trend at the protein level. Further comparative analysis of clinicopathological characteristics based on DPF2 immunohistochemical scores was conducted in 60 patients with colon adenocarcinoma. The results showed that high DPF2 expression was significantly associated with more advanced TNM stage (*P* = 0.007) and lymph node metastasis (*P* = 0.009) (Table 1). No statistically significant differences were observed between the two groups in terms of age, sex, differentiation status, or recurrence. At the last follow-up, the survival proportion of patients in the high-expression group was lower than that in the low-expression group (29.6% vs. 60.6%, *P* = 0.021), indicating that high DPF2 expression was associated with adverse clinical outcomes. Kaplan–Meier survival analysis further showed that the overall survival probability of patients in the high-DPF2 expression group was significantly lower than that in the low-expression group (log-rank test, *P* = 0.0078) (Figure 3D), further supporting the association between high DPF2 expression and poor prognosis.

**TABLE 1:** Clinicopathological characteristics according to DPF2 expression in patients with colon adenocarcinoma.

Variable	Total (n = 60)	Low expression (n = 33)	High expression (n = 27)	P value
Age, years, median (IQR)	59.5 (54.0–70.0)	65.0 (58.0–70.0)	58.0 (52.0–64.0)	0.090
**Sex, n (%)**	​	​	​	1.000
Male	33 (55.0%)	18 (54.5%)	15 (55.6%)	​
Female	27 (45.0%)	15 (45.5%)	12 (44.4%)	​
**Differentiation, n (%)**	​	​	​	0.084
Well differentiated	17 (28.3%)	8 (24.2%)	9 (33.3%)	​
Moderately differentiated	34 (56.7%)	17 (51.5%)	17 (63.0%)	​
Poorly differentiated	9 (15.0%)	8 (24.2%)	1 (3.7%)	​
**TNM stage, n (%)**	​	​	​	**0.007**
Stage I	11 (18.3%)	9 (27.3%)	2 (7.4%)	​
Stage II	17 (28.3%)	13 (39.4%)	4 (14.8%)	​
Stage III	22 (36.7%)	8 (24.2%)	14 (51.9%)	​
Stage IV	10 (16.7%)	3 (9.1%)	7 (25.9%)	​
**Lymph node metastasis, n (%)**	​	​	​	**0.009**
No	32 (53.3%)	23 (69.7%)	9 (33.3%)	​
Yes	28 (46.7%)	10 (30.3%)	18 (66.7%)	​
**Distant metastasis, n (%)**	​	​	​	0.097
No	50 (83.3%)	30 (90.9%)	20 (74.1%)	​
Yes	10 (16.7%)	3 (9.1%)	7 (25.9%)	​
**Recurrence, n (%)**	​	​	​	0.397
No	42 (70.0%)	25 (75.8%)	17 (63.0%)	​
Yes	18 (30.0%)	8 (24.2%)	10 (37.0%)	​
**Vital status at last follow-up, n (%)**	​	​	​	**0.021**
Alive	28 (46.7%)	20 (60.6%)	8 (29.6%)	​
Dead	32 (53.3%)	13 (39.4%)	19 (70.4%)	​

Bold values indicate statistical significance (P < 0.05). Continuous variables are presented as median (interquartile range, IQR) and were compared using the Mann–Whitney U test. Categorical variables are presented as n (%), with percentages calculated within each column unless otherwise indicated, and were compared using Pearson’s chi-square test or Fisher’s exact test, as appropriate. TNM, tumor–node–metastasis; IQR, interquartile range.

### 3.3 Stratified analysis across immune cell subsets revealed cell-type-specific risk effects of DPF2

**FIGURE 4:**
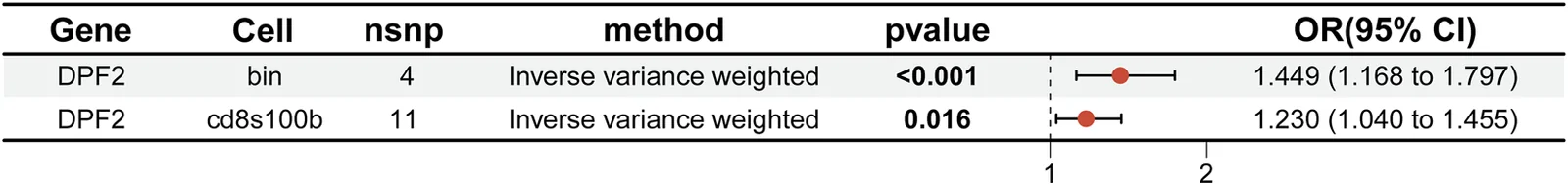
Association of DPF2 with colon adenocarcinoma risk across immune cell subsets. Results of single-cell Mendelian randomization analyses evaluating the associations between genetically predicted DPF2 expression in different immune cell subsets and the risk of colon adenocarcinoma. The strongest risk-associated signal was observed in TCL1A^+^ FCER2^+^ B cells, whereas CD8^+^ S100B^+^ T cells showed a suggestive positive association.

Given the critical role of the tumor immune microenvironment in colon adenocarcinoma progression, we further assessed the association between genetically predicted DPF2 expression and colon adenocarcinoma risk across 14 immune cell subsets based on single-cell expression quantitative trait loci (sc-eQTL) data. The results showed that in TCL1A^+^ FCER2^+^ B cells (bin), increased genetically predicted DPF2 expression was significantly associated with an increased risk of colon adenocarcinoma. IVW analysis yielded an OR of 1.449 (95% CI 1.168–1.797, *P* < 0.001, nsnp = 4). This association remained significant after Bonferroni correction for 14 immune cell subsets, suggesting that bin cells may represent the principal cellular carrier of the DPF2-associated risk effect. In addition, a positive association was also observed in CD8^+^ S100B^+^ T cells (cd8s100b), with IVW analysis showing an OR of 1.230 (95% CI 1.040–1.455, *P* = 0.016, nsnp = 11) (Figure 4); however, this result did not survive correction for multiple testing and should therefore be considered a suggestive signal. No stable significant associations were observed in the remaining immune cell subsets (Supplementary Table S5). Collectively, these findings indicate that the effect of DPF2 on colon adenocarcinoma risk is unlikely to reflect the average effect across all immune cells, but rather appears to exhibit clear immune cell type specificity, with the strongest evidence observed in TCL1A^+^ FCER2^+^ B cells.

### 3.4 Single-cell expression validation revealed a heterogeneous distribution of DPF2 in the CRC immune microenvironment

**FIGURE 5:**
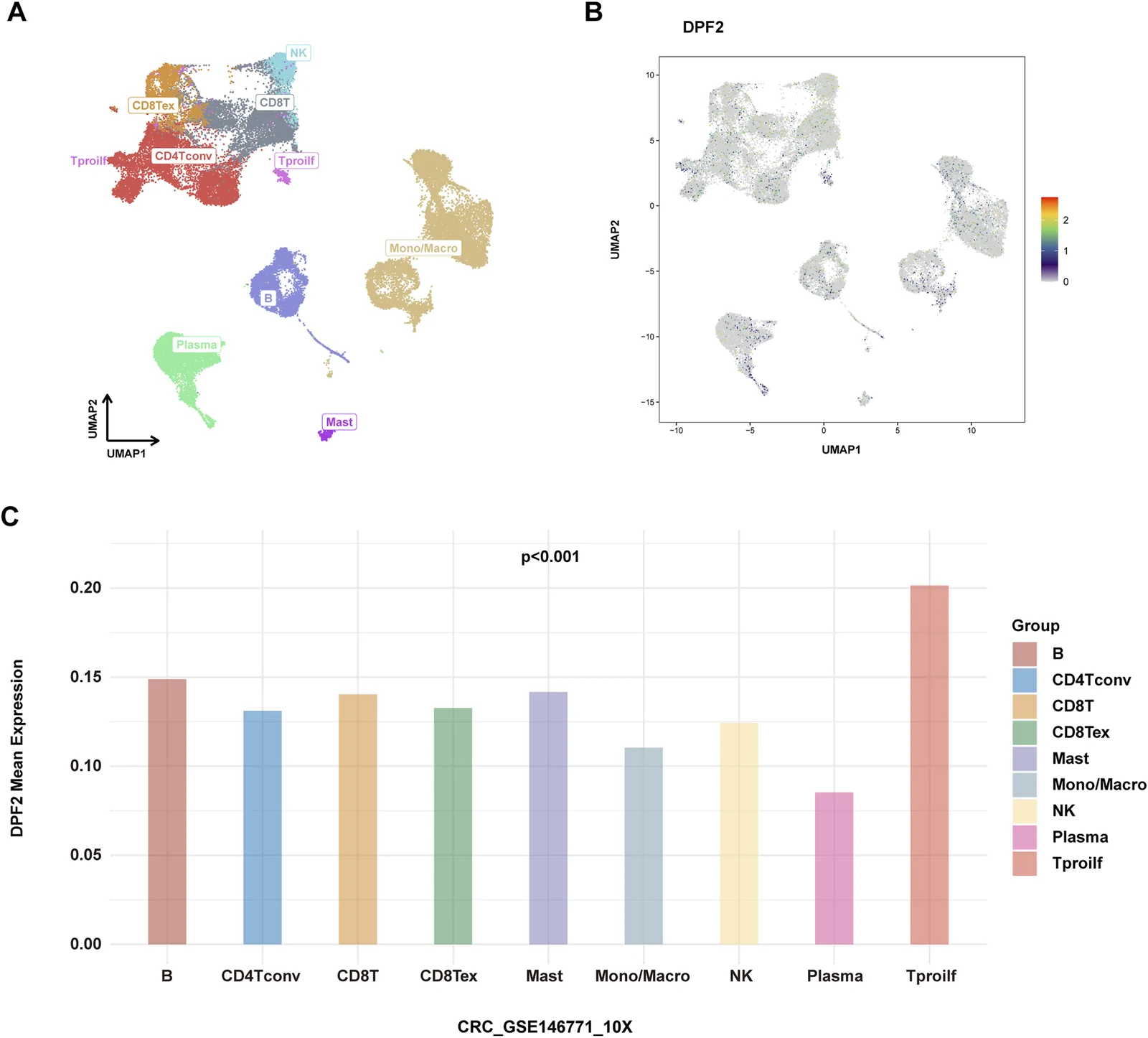
Single-cell transcriptomic analysis reveals heterogeneous DPF2 expression in the colorectal cancer immune microenvironment. **(A)** UMAP clustering of the major immune cell populations in the GSE146771 dataset. **(B)** UMAP visualization of DPF2 expression in the single-cell transcriptomic dataset. **(C)** Comparison of the average DPF2 expression levels among different immune cell populations, showing significant differences across cell types (P < 0.001).

To provide complementary support for the cell-type-specific analysis results at the transcriptomic level, we further examined the expression distribution of DPF2 in CRC single-cell transcriptomic data. UMAP dimensionality reduction showed that major immune cell populations, including B cells, CD4Tconv, CD8T, CD8Tex, NK cells, Plasma cells, Mono/Macro cells, Mast cells, and proliferating T cells (Tprolif), formed relatively distinct clusters in two-dimensional space, indicating that this dataset effectively distinguished the major immune cell lineages within the CRC immune microenvironment (Figure 5A).

Single-cell visualization of DPF2 expression demonstrated an obviously heterogeneous distribution across immune cell populations rather than uniform expression. The strongest expression signal was observed in Tprolif cells, while DPF2 expression was also detectable in B cells, CD8T cells, and CD8Tex cells (Figure 5B). Further summary of average expression by cell type showed that DPF2 had the highest average expression in Tprolif cells, followed by B cells; Mast cells, CD8T cells, CD8Tex cells, and CD4Tconv cells exhibited comparable intermediate expression levels, whereas Plasma cells showed the lowest expression. Overall differences in DPF2 expression among cell groups were statistically significant (Kruskal–Wallis test, *P* < 0.001; Figure 5C).

It should be noted that single-cell expression analysis reflects the relative expression patterns of DPF2 across different immune cell populations and cannot directly represent the strength of the causal effect of DPF2 expression changes in a given cell subset on disease risk. Nevertheless, the detectable expression of DPF2 in both B cells and CD8-related cell populations, together with the aforementioned scMR results, supports at the transcriptomic level the notion that DPF2 may participate in colon adenocarcinoma development through specific immune cell subsets.

### 3.5 Spatial transcriptomic analysis revealed preferential expression of DPF2 in tumor regions

**FIGURE 6:**
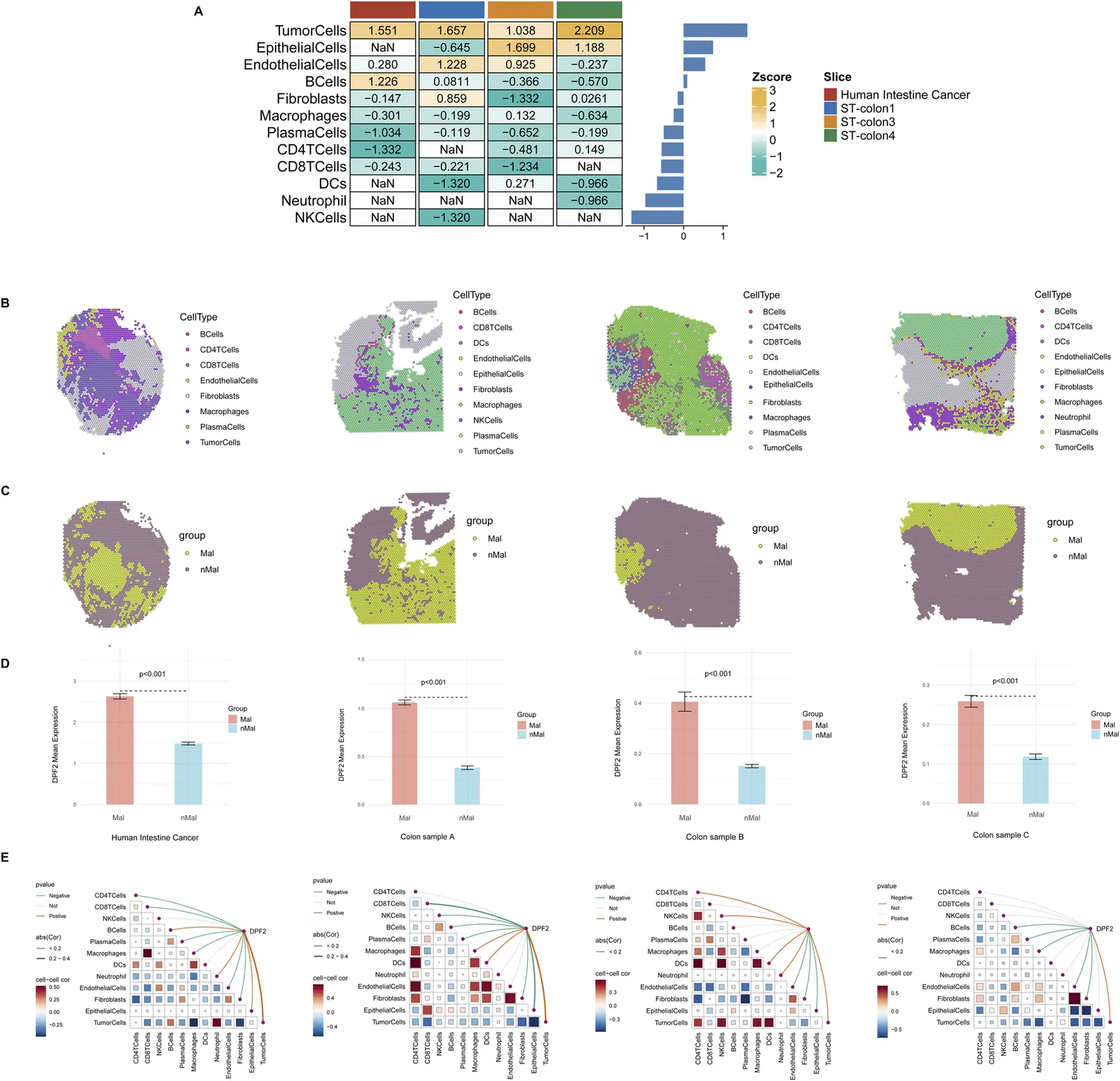
Spatial transcriptomic analysis shows preferential enrichment of DPF2 in tumor cell-dominant regions of colorectal cancer tissues. **(A)** Heatmap showing the relative expression of DPF2 across regions dominated by different cell types in representative sections. **(B)** Spatial distribution of the dominant cell types in each section. **(C)** Spatial distribution of malignant regions (Mal) and non-malignant regions (nMal), defined according to the proportion of TumorCells. **(D)** Comparison of the average DPF2 expression levels between Mal and nMal regions in each section. **(E)** Integrated correlation analysis of DPF2 expression with the proportions of different cell types and with inter-cell-type relationships.

To further evaluate the spatial localization of DPF2 expression and its relationship with local microenvironmental composition in the tissue context, we analyzed four representative colorectal cancer spatial transcriptomic sections. The results showed that DPF2 was overall highly expressed in the selected samples and was generally enriched in tumor-associated regions. When summarized according to dominant cell type, tumor cell-dominated regions consistently showed the highest relative DPF2 expression across all four sections, suggesting a stable association between DPF2 and tumor cell-enriched microenvironments (Figure 6A). In addition to tumor cell-dominated areas, some expression was also observed in regions dominated by Epithelial Cells, Endothelial Cells, or Fibroblasts in individual samples.

Spatial mapping of dominant cell types further demonstrated relatively clear tissue compartmentalization within each section, with different regions dominated by tumor cells, immune cells, or stromal-associated cells, although the specific composition patterns varied across samples (Figure 6B). On this basis, malignant regions (Mal) and non-malignant regions (nMal), defined according to the proportion of TumorCells, formed relatively distinct spatial boundaries in each section (Figure 6C).

Quantitative comparison between Mal and nMal regions showed that the average expression of DPF2 was significantly higher in malignant regions than in non-malignant regions across all four sections (all *P* < 0.001) (Figure 6D). Further correlation analysis indicated that DPF2 expression was positively correlated with the proportion of TumorCells in all sections, whereas associations with other cellular components varied somewhat among samples (Figure 6E). Overall, the relationship between DPF2 and the tumor cell component was the most stable. These results indicate that DPF2 is not uniformly distributed within colorectal cancer tissues, but is preferentially concentrated in tumor cell-dominant regions, further supporting its close association with colorectal cancer development and progression from a spatial perspective.

### 3.6 Systematic screening identified metabolites associated with colon adenocarcinoma risk

**FIGURE 7:**
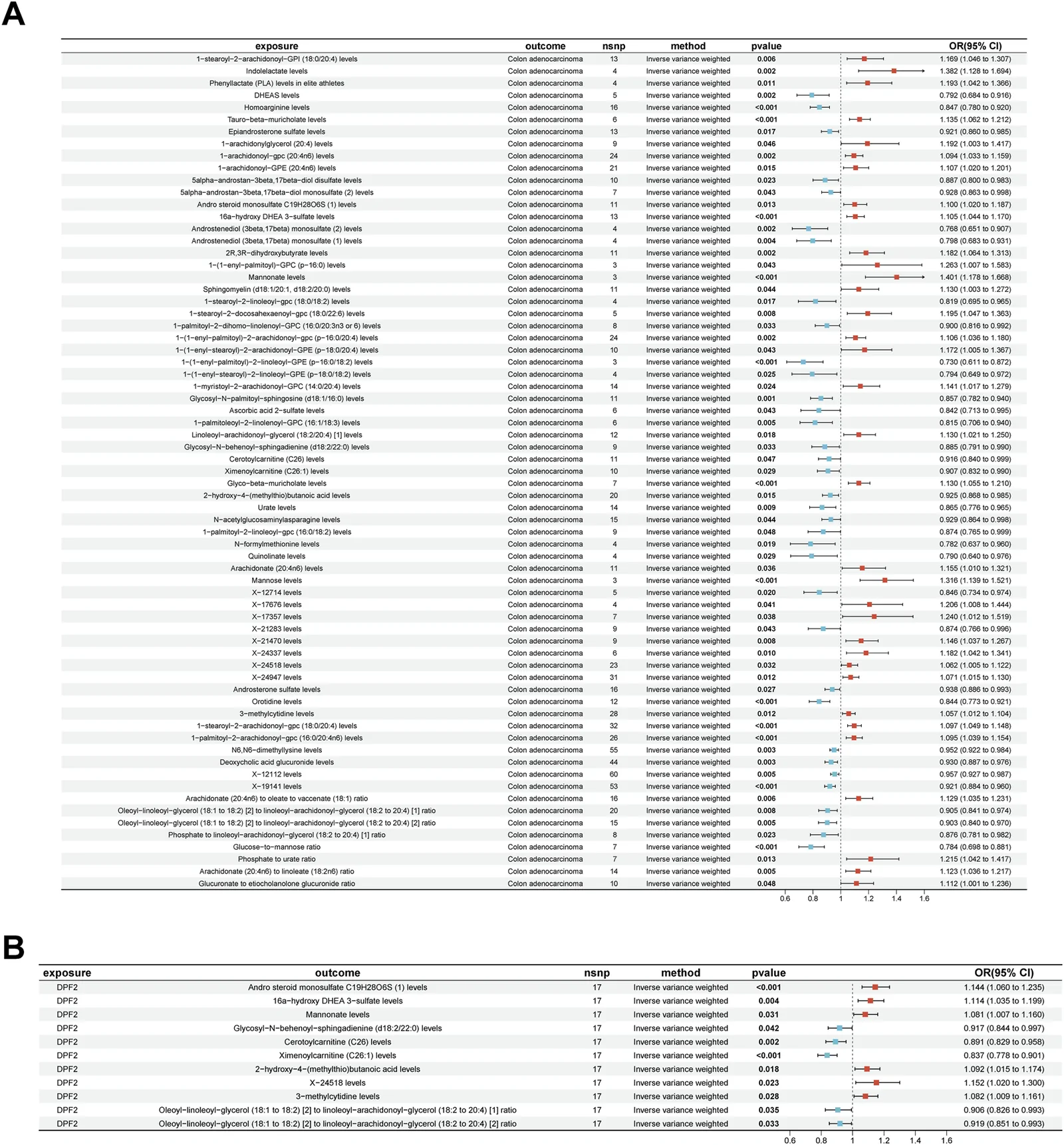
Circulating metabolites associated with colon adenocarcinoma risk and their associations with DPF2. **(A)** Screening results of circulating metabolites significantly associated with colon adenocarcinoma risk. **(B)** Association analysis between DPF2 and metabolites related to colon adenocarcinoma.

To further explore potential metabolic pathways associated with colon adenocarcinoma, we first performed a systematic screening of circulating metabolites related to colon adenocarcinoma at the metabolomic level. Based on large-scale circulating metabolite GWAS data, we conducted two-sample MR analyses between all eligible metabolites and colon adenocarcinoma risk. The results identified 69 metabolites showing statistical associations with colon adenocarcinoma risk, covering multiple categories, including lipid metabolites, acylcarnitines, steroid hormone/endocrine-related metabolites, and various metabolite ratios (Figure 7A; Supplementary Table S6). These findings suggest that the development and progression of colon adenocarcinoma may be accompanied by extensive metabolic reprogramming.

### 3.7 Causal association analysis between DPF2 and colon adenocarcinoma-related metabolites

After obtaining the set of colon adenocarcinoma-related metabolites, we further evaluated the potential impact of genetically predicted DPF2 expression on these metabolites. The results showed that DPF2 was significantly genetically associated with 11 metabolites, suggesting that DPF2 may participate in colon adenocarcinoma progression through multiple metabolic pathways (Figure 7B; Supplementary Table S7).

Specifically, DPF2 was negatively associated with Ximenoylcarnitine (C26:1), and this metabolite was also negatively associated with colon adenocarcinoma risk at the genetic level, suggesting that it may represent a protective lipid metabolic signal. In contrast, DPF2 was positively associated with 16α-hydroxy DHEA 3-sulfate and Andro steroid monosulfate C19H28O6S (1), both of which were genetically associated with increased colon adenocarcinoma risk, suggesting that DPF2 may promote tumor progression through steroid/endocrine-related metabolic axes.

### 3.8 Mediation analysis of the DPF2–metabolite–colon adenocarcinoma axis

**FIGURE 8:**
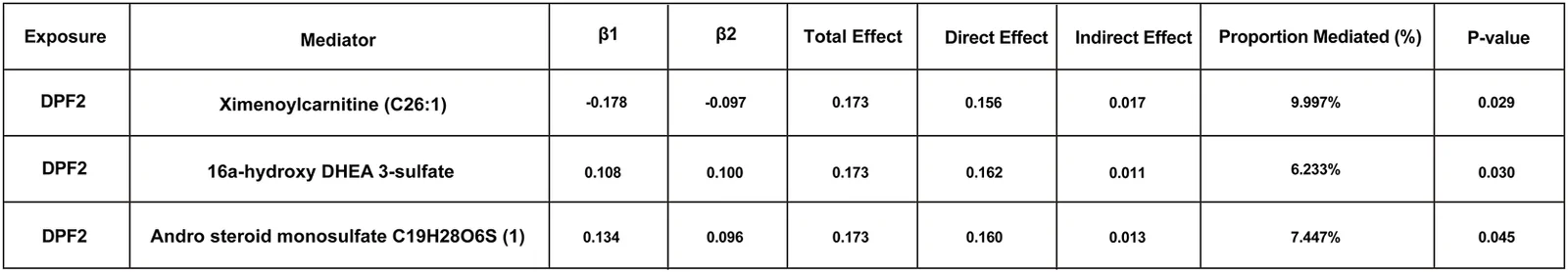
Mediation analysis of the effects of DPF2 on colon adenocarcinoma risk through candidate metabolites. Two-step mediation analyses showing that the effect of DPF2 on colon adenocarcinoma risk was partially mediated through Ximenoylcarnitine (C26:1), 16α-hydroxy DHEA 3-sulfate, and Andro steroid monosulfate.

To clarify the specific pathways through which DPF2 influences colon adenocarcinoma risk via metabolic pathways, we further performed mediation analysis under a two-step MR framework (Figure 8). The results showed that DPF2 could indirectly increase colon adenocarcinoma risk by suppressing Ximenoylcarnitine (C26:1) levels (*P* = 0.0294), with a mediation proportion of approximately 9.99%. In light of the directionality of the results, this finding suggests that DPF2 may partly mediate its risk-promoting effect by weakening a potentially protective lipid metabolic signal.

In addition, DPF2 could indirectly increase colon adenocarcinoma risk by elevating the levels of 16α-hydroxy DHEA 3-sulfate and Andro steroid monosulfate C19H28O6S (1), with corresponding mediation effects of *P* = 0.0304 and mediation proportion = 6.23%, and *P* = 0.0445 and mediation proportion = 7.45%, respectively. These results collectively suggest that the effect of DPF2 on colon adenocarcinoma risk is not entirely direct, but may be partly mediated through lipid metabolism and steroid hormone-related metabolic pathways.

## 4 Discussion

To our knowledge, this is the first study to systematically investigate, from a causal inference perspective and at both population-genetic and single-cell resolution, the immune and metabolic regulatory characteristics of crotonylation-related genes in the development and progression of colon adenocarcinoma (COAD), highlighting DPF2 as a core crotonylation reader that is genetically associated with COAD risk and progression. Based on Mendelian randomization and integrating single-cell eQTL data, GEO-based expression analyses, and metabolite genetic association analyses, this study constructed a DPF2-centered epigenetic-metabolic framework with immune cell type specificity, thereby providing new evidence for understanding the molecular mechanisms underlying COAD risk. Furthermore, we incorporated supplementary evidence from public single-cell and spatial transcriptomic datasets to cross-validate the expression pattern of DPF2 from the perspectives of cell-subset resolution and tissue spatial localization. Importantly, these single-cell and spatial transcriptomic findings should be interpreted as supportive expression evidence in the broader CRC context, rather than as direct causal inference results specific to COAD. Taken together, the present study integrates COAD-focused causal inference with CRC-context supportive transcriptomic evidence, and thereby establishes a multilayered evidence chain for DPF2 characterized by genetic association, aberrant expression, cell-type-restricted signals, and partial metabolic mediation.

Among the 16 initially screened crotonylation-related genes, our study clearly identified DPF2 as being significantly positively associated with colon adenocarcinoma risk, suggesting its potential driver role in tumor pathogenesis. Importantly, this finding does not imply that other crotonylation-related genes lack biological function, but more likely reflects differences in detectability at the genetic level among distinct regulators. Lysine crotonylation (Kcr) is a highly dynamic post-translational modification whose occurrence directly depends on the intracellular supply of short-chain acyl-coenzyme A (crotonyl-CoA) (Yuan et al., 2023), making it highly sensitive to fluctuations in lipid metabolism, amino acid metabolism, and overall metabolic status. Previous studies have shown that Kcr is not a stable structural epigenetic mark in the traditional sense, but rather acts as a chromatin modification that rapidly translates metabolic changes into transcriptional activation signals (Sabari et al., 2017; Yang et al., 2023). Therefore, the biological effects of Kcr largely depend on whether it is captured by specific reader proteins and converted into transcriptional outputs with sustained biological consequences. As one of the few currently well-characterized crotonylation readers, DPF2 can selectively bind crotonylated sites on histone H3 via its tandem PHD domains, thereby regulating chromatin accessibility and remodeling downstream transcriptional programs (Xiong et al., 2016). Compared with crotonylation “writers” or “erasers,” DPF2 occupies a position closer to the terminal node of transcriptional regulation, a functional property that may make it more likely to exhibit stable and reproducible disease risk effects at the population-genetic level. Previous studies have shown that crotonylation-mediated transcriptional regulation is not restricted to a single tumor type, and that aberrantly elevated crotonylation levels are closely associated with malignant progression in several cancers, including lung cancer, cervical cancer, and hepatocellular carcinoma (Yang et al., 2023; Wang et al., 2025). However, the functional consequences of Kcr differ substantially across cell types and physiological contexts (Sabari et al., 2017; Yang et al., 2023). Combined with the immune cell type-specific causal effects observed at single-cell resolution in this study, DPF2-mediated crotonylation regulation may act as a key threshold regulator of metabolism–epigenetics coupling in specific immune cell subsets, thereby driving the reprogramming of cellular functional states under conditions of metabolic or immune microenvironmental change and ultimately influencing COAD risk. Thus, through genetic causal inference, our study provides new evidence supporting the role of DPF2, a key reader node within the crotonylation regulatory system, in colon adenocarcinoma risk, thereby advancing the understanding of the causal role of metabolism-coupled epigenetic regulation in tumor development.

Notably, this study innovatively incorporated single-cell eQTL data to explore the impact of DPF2 expression in immune cell subsets on colon adenocarcinoma risk. We found that genetically predicted high DPF2 expression in a specific B-cell subset (TCL1A^+^ FCER2^+^ B cells) showed the strongest risk-associated signal, while a certain degree of association was also observed in CD8^+^ S100B^+^ T cells. These findings suggest that the immune regulatory effects associated with DPF2 may be mediated primarily through specific B-cell subsets rather than being universally present across all immune cells. Previous studies have demonstrated marked functional heterogeneity among tumor-infiltrating B cells in solid tumors, with distinct subsets exerting different or even opposing roles in antigen presentation, antibody responses, and immune regulation (Shalapour et al., 2015; Affara et al., 2014). Some B-cell subsets may exert antitumor effects by promoting tertiary lymphoid structure formation and enhancing T-cell responses, whereas other immunoregulatory B-cell populations may suppress effector T-cell function and promote immune evasion through the secretion of immunosuppressive factors such as IL-10 and TGF-β (Shalapour et al., 2015). Single-cell transcriptomic studies of human tumor samples further support the notion that tumor-infiltrating B cells are highly heterogeneous in transcriptional states and functional programs and are closely associated with tumor progression and clinical outcomes (Yang et al., 2024c). Immune landscape studies in CRC have likewise shown that the composition and functional state of B cells are closely related to tumor stage, immune phenotype, and therapeutic response, and constitute important determinants of the tumor immune ecosystem (Ferkel et al., 2025; Fridman et al., 2022). Therefore, the genetic association signal observed in this study for DPF2 in a specific B-cell subset suggests that DPF2 may influence B-cell functional polarization or immunoregulatory programs through epigenetic regulatory mechanisms, thereby amplifying risk-promoting immune effects within the tumor immune microenvironment. It should be emphasized that this study did not directly demonstrate the specific molecular mechanisms of DPF2 in B cells, but instead provides supportive evidence for its potential role in CRC immune regulation from the perspective of genetic causal inference. By contrast, the association of DPF2 in the CD8^+^ T-cell subset did not survive correction for multiple comparisons and should therefore be regarded as suggestive. Previous studies have shown that tumor-associated CD8^+^ T cells do not constitute a single homogeneous population, but instead encompass effector, memory, and multiple stages of exhausted phenotypes, all of which are shaped by complex transcriptional and epigenetic regulatory networks (Wherry and Kurachi, 2015; McLane et al., 2019). For example, CD8^+^ T cells play a central role in antitumor immunity, but under chronic inflammation and in the tumor context they may acquire an exhausted functional state. The potential role of DPF2 in CD8^+^ T-cell subsets suggests that it may further aggravate tumor progression by inducing shifts in T-cell functional states or exhausted phenotypes. Therefore, whether DPF2 exerts regulatory roles in specific CD8^+^ T-cell functional states requires further validation in larger single-cell genetic regulatory datasets and in follow-up functional experiments. In addition, our supplementary single-cell transcriptomic analysis showed that DPF2 is not uniformly expressed in the CRC immune microenvironment, but instead exhibits clear expression differences across immune cell groups; spatial transcriptomic analysis further showed that DPF2 is more enriched in malignant regions dominated by tumor cells at the tissue level. These findings are not inconsistent with the scMR results, but rather provide complementary support for the tumor relevance and cell-type-restricted effects of DPF2 from the two perspectives of cellular expression distribution and tissue spatial localization.

Through systematic metabolite MR analysis, this study identified significant associations between DPF2 and multiple metabolites, particularly the lipid metabolite Ximenoylcarnitine (C26:1) and steroid hormone-related metabolites. These findings suggest that the pathogenic effects of DPF2 may not be mediated through a single metabolic axis, but rather through the concerted involvement of multiple metabolic pathways in COAD development and progression, implying that DPF2 may function as an upstream integrative node in the metabolism–epigenetics regulatory network. Previous studies have shown that tumor metabolic reprogramming is not only a consequence of the energetic and biosynthetic demands of tumor cells, but also a key factor in shaping the tumor immune microenvironment, with dysregulated lipid metabolism being considered one of the major drivers of progression and immune evasion in multiple solid tumors (Pavlova et al., 2022; Snaebjornsson et al., 2020). Fatty acid oxidation and related metabolic intermediates not only provide a continuous energy supply for tumor cells, but also regulate tumor–immune interactions by affecting membrane composition, signal transduction, and the metabolic adaptability of immune cells (Pavlova et al., 2022; Koundouros and Poulogiannis, 2020). In this study, DPF2 indirectly increased COAD risk by decreasing Ximenoylcarnitine levels, suggesting that it may partially mediate its risk-promoting effect by weakening a potentially protective lipid metabolic signal. In addition, we observed positive causal associations between DPF2 and multiple steroid hormones and their sulfated derivatives, further supporting the possibility that DPF2 regulates tumor progression through the endocrine–immune–metabolic axis. Recent studies have shown that steroid hormone metabolism not only regulates tumor cell proliferation and differentiation through classical nuclear receptor signaling pathways, but can also indirectly influence the tumor immune microenvironment by reshaping immune cell metabolic states and transcriptional programs (Anderson and Acharya, 2022; Bhattacharyya et al., 2025; Smith et al., 2025). In previous CRC-related studies, endocrine-related metabolic axes have been proposed to participate in disease progression by affecting tumor cell proliferative potential, the degree of immunosuppression, and inflammatory status (Rodríguez-Santiago et al., 2024). The finding that DPF2 promotes the levels of these metabolites further supports the possibility that it may regulate tumor progression through steroid hormone signaling. Notably, our mediation analysis showed that lipid-related metabolites and steroid hormone-related metabolites each explained approximately 6%–10% of the total effect between DPF2 and COAD risk. This mediation proportion suggests that these metabolic pathways play partial mediating roles in the risk-related effects mediated by DPF2, which is consistent with the biological nature of COAD as a multifactorial disease and with the concept that epigenetic regulation usually exerts its effects through multiple parallel metabolic pathways. It should be emphasized that the causal inference in this study was based on circulating metabolite levels and therefore cannot fully reflect metabolic alterations within the tumor local environment or within immune cells; the underlying mechanisms still require validation using tissue-level metabolomics and functional experiments.

This study has several methodological strengths. First, the use of Mendelian randomization helps reduce confounding bias and reverse causality that commonly affect conventional observational studies, allowing a more robust evaluation of the potential association between DPF2 expression and COAD risk. Second, this study introduced genetically regulated single-cell transcriptomic data into the MR analytical framework. Compared with conventional MR analyses based on bulk transcriptomic data, single-cell MR enables the dissection of potential gene expression effects at a scale closer to true biological functional units. If only bulk data were used, signals with opposite directions or substantially different effect sizes across immune cell subsets could be averaged out, thereby masking critical cell-type-specific associations (Hao et al., 2024). In recent years, multiple high-throughput single-cell genetic regulatory studies have confirmed that the genetic effects on gene expression are strongly dependent on cell state and cell type (Sun et al., 2025; Ray et al., 2025), and our study further supports the necessity and potential value of applying the scMR framework in tumor immunology research. In addition, by integrating multi-omics data to construct a multilayered genetic–immune–metabolic analytical framework, this study enhanced the overall coherence and biological interpretability of the conclusions. More importantly, the incorporation of both single-cell transcriptomic and spatial transcriptomic evidence allowed the genetic risk signals to be supported by complementary evidence from both cellular distribution and tissue localization, further strengthening the biological interpretability of the findings.

Several limitations of this study should also be acknowledged and addressed in future work. First, the data used in this study were derived mainly from European populations, and the generalizability of the findings to other ethnic and geographic populations remains to be validated. Second, some datasets used in this study originated from public databases and may therefore be subject to potential limitations such as differences in data standardization and measurement error. In addition, although Mendelian randomization provides robust statistical evidence, this study was based on genetically predicted gene expression levels and therefore cannot directly reflect dynamic changes in DPF2 protein abundance or in the levels of crotonylation modifications mediated by DPF2. Furthermore, the metabolite analyses were based on circulating-level data and cannot fully represent metabolic states within tumor cells or immune cells. The specific molecular mechanisms still require further validation and confirmation through functional studies in cellular and animal models.

In summary, this study provides support at both the population-genetic and single-cell levels for the potential importance of DPF2 in the development and progression of colon adenocarcinoma and suggests that DPF2 may jointly contribute to tumor progression through specific immune cell subsets and metabolic pathways. These findings offer a new perspective for understanding the molecular pathological mechanisms of COAD, while public single-cell and spatial transcriptomic data from the broader CRC context provide complementary supportive evidence regarding the expression distribution and spatial localization of DPF2. Future studies using cellular and animal models are still needed to further validate the biological roles and translational relevance of DPF2-related pathways in tumor biology.

## Data Availability

The datasets presented in this study can be found in online repositories. The names of the repository/repositories and accession number(s) can be found below: https://www.ncbi.nlm.nih.gov/geo/, GSE4183; GSE8671; GSE20916; GSE32323; GSE37364; GSE146771 https://portal.gdc.cancer.gov/, TCGA-COAD https://www.ebi.ac.uk/gwas/, GCST90199640-GCST90201019 https://finngen.gitbook.io/documentation/, finngen_R12_C3_COLON_ADENO_EXALLC https://www.eqtlgen.org/cis-eqtls.html, eQTLGen cis-eQTL data.

## Supplementary material

SUPPLEMENTARY FIGURE S1 Flowchart of the intersection analysis between the eQTLGen database and the crotonylation-related gene set.

## References

[B1] Affara N. I. Ruffell B. Medler T. R. Gunderson A. J. Johansson M. Bornstein S. (2014). B cells regulate macrophage phenotype and response to chemotherapy in squamous carcinomas. Cancer Cell 25 (6), 809–821. 10.1016/j.ccr.2014.04.026 24909985 PMC4063283

[B2] Anderson A. C. Acharya N. (2022). Steroid hormone regulation of immune responses in cancer. Immunometabolism (Cobham) 4 (4), e00012. 10.1097/in9.0000000000000012 36337733 PMC9622373

[B3] Bhattacharyya T. Das P. Banerjee R. (2025). Targeting steroid hormone receptors for anti-cancer therapy. Vitam. Horm. 129, 1–59. 10.1016/bs.vh.2024.10.002 40812943

[B4] Binnewies M. Roberts E. W. Kersten K. Chan V. Fearon D. F. Merad M. (2018). Understanding the tumor immune microenvironment (TIME) for effective therapy. Nat. Med. 24 (5), 541–550. 10.1038/s41591-018-0014-x 29686425 PMC5998822

[B5] Bray F. Laversanne M. Sung H. Ferlay J. Siegel R. L. Soerjomataram I. (2024). Global cancer statistics 2022: GLOBOCAN estimates of incidence and mortality worldwide for 36 cancers in 185 countries. CA Cancer J. Clin. 74 (3), 229–263. 10.3322/caac.21834 38572751

[B6] Chen Y. Lu T. Pettersson-Kymmer U. Stewart I. D. Butler-Laporte G. Nakanishi T. (2023). Genomic atlas of the plasma metabolome prioritizes metabolites implicated in human diseases. Nat. Genet. 55 (1), 44–53. 10.1038/s41588-022-01270-1 36635386 PMC7614162

[B7] Chen Y. Wang D. Li Y. Qi L. Si W. Bo Y. (2024). Spatiotemporal single-cell analysis decodes cellular dynamics underlying different responses to immunotherapy in colorectal cancer. Cancer Cell 42 (7), 1268–1285.e7. 10.1016/j.ccell.2024.06.009 38981439

[B8] Chu X. Li X. Zhang Y. Dang G. Miao Y. Xu W. (2024). Integrative single-cell analysis of human colorectal cancer reveals patient stratification with distinct immune evasion mechanisms. Nat. Cancer 5 (9), 1409–1426. 10.1038/s43018-024-00807-z 39147986

[B9] Fedchenko N. Reifenrath J. (2014). Different approaches for interpretation and reporting of immunohistochemistry analysis results in the bone tissue - a review. Diagn Pathol. 9, 221. 10.1186/s13000-014-0221-9 25432701 PMC4260254

[B10] Ferkel S. A. M. Holman E. A. Sojwal R. S. Rubin S. J. S. Rogalla S. (2025). Tumor-infiltrating immune cells in colorectal cancer. Neoplasia 59, 101091. 10.1016/j.neo.2024.101091 39642846 PMC11665540

[B11] Fridman W. H. Meylan M. Petitprez F. Sun C. M. Italiano A. Sautès-Fridman C. (2022). B cells and tertiary lymphoid structures as determinants of tumour immune contexture and clinical outcome. Nat. Rev. Clin. Oncol. 19 (7), 441–457. 10.1038/s41571-022-00619-z 35365796

[B12] Han F. Shen W. Zhang X. Du M. Ye Q. Ma J. (2025). Protein crotonylation in cancer: mechanisms, functions, and therapeutic potential. Cell Biol. Toxicol. 42, 10. 10.1007/s10565-025-10130-7 41351798 PMC12789249

[B13] Hao R. H. Zhang T. P. Jiang F. Liu J. H. Dong S. S. Li M. (2024). Revealing brain cell-stratified causality through dissecting causal variants according to their cell-type-specific effects on gene expression. Nat. Commun. 15 (1), 4890. 10.1038/s41467-024-49263-4 38849352 PMC11161590

[B14] Jiang G. Li C. Lu M. Lu K. Li H. (2021). Protein lysine crotonylation: past, present, perspective. Cell Death Dis. 12 (7), 703. 10.1038/s41419-021-03987-z 34262024 PMC8280118

[B15] Koundouros N. Poulogiannis G. (2020). Reprogramming of fatty acid metabolism in cancer. Br. J. Cancer 122 (1), 4–22. 10.1038/s41416-019-0650-z 31819192 PMC6964678

[B16] Li Q. Geng S. Luo H. Wang W. Mo Y. Q. Luo Q. (2024). Signaling pathways involved in colorectal cancer: pathogenesis and targeted therapy. Signal Transduct. Target Ther. 9 (1), 266. 10.1038/s41392-024-01953-7 39370455 PMC11456611

[B17] Ling A. Löfgren-Burström A. Larsson P. Li X. Wikberg M. L. Öberg Å. (2017). TAP1 down-regulation elicits immune escape and poor prognosis in colorectal cancer. Oncoimmunology 6 (11), e1356143. 10.1080/2162402X.2017.1356143 29147604 PMC5674960

[B18] Mas G. Man N. Nakata Y. Martinez-Caja C. Karl D. Beckedorff F. (2023). The SWI/SNF chromatin-remodeling subunit DPF2 facilitates NRF2-dependent antiinflammatory and antioxidant gene expression. J. Clin. Invest 133 (13), e158419. 10.1172/jci158419 37200093 PMC10313367

[B19] McLane L. M. Abdel-Hakeem M. S. Wherry E. J. (2019). CD8 T cell exhaustion during chronic viral infection and cancer. Annu. Rev. Immunol. 37, 457–495. 10.1146/annurev-immunol-041015-055318 30676822

[B20] Morgan E. Arnold M. Gini A. Lorenzoni V. Cabasag C. J. Laversanne M. (2023). Global burden of colorectal cancer in 2020 and 2040: incidence and mortality estimates from GLOBOCAN. Gut 72 (2), 338–344. 10.1136/gutjnl-2022-327736 36604116

[B21] Nicolini A. Ferrari P. (2024). Involvement of tumor immune microenvironment metabolic reprogramming in colorectal cancer progression, immune escape, and response to immunotherapy. Front. Immunol. 15, 1353787. 10.3389/fimmu.2024.1353787 39119332 PMC11306065

[B22] Pavlova N. N. Zhu J. Thompson C. B. (2022). The hallmarks of cancer metabolism: still emerging. Cell Metab. 34 (3), 355–377. 10.1016/j.cmet.2022.01.007 35123658 PMC8891094

[B23] Pelka K. Hofree M. Chen J. H. Sarkizova S. Pirl J. D. Jorgji V. (2021). Spatially organized multicellular immune hubs in human colorectal cancer. Cell 184 (18), 4734–4752.e20. 10.1016/j.cell.2021.08.003 34450029 PMC8772395

[B24] Ray A. Alabarse P. Malik R. Sargurupremraj M. Bernhagen J. Dichgans M. (2025). Single-cell transcriptome-wide Mendelian randomization and colocalization analyses uncover cell-specific mechanisms in atherosclerotic cardiovascular disease. Am. J. Hum. Genet. 112 (7), 1597–1609. 10.1016/j.ajhg.2025.06.001 40555237 PMC12256824

[B25] Rodríguez-Santiago Y. Garay-Canales C. A. Nava-Castro K. E. Morales-Montor J. (2024). Sexual dimorphism in colorectal cancer: molecular mechanisms and treatment strategies. Biol. Sex. Differ. 15 (1), 48. 10.1186/s13293-024-00623-1 38867310 PMC11170921

[B26] Sabari B. R. Zhang D. Allis C. D. Zhao Y. (2017). Metabolic regulation of gene expression through histone acylations. Nat. Rev. Mol. Cell Biol. 18 (2), 90–101. 10.1038/nrm.2016.140 27924077 PMC5320945

[B27] Sanderson E. Glymour M. M. Holmes M. V. Kang H. Morrison J. Munafò M. R. (2022). Mendelian randomization. Nat. Rev. Methods Prim. 2 (1), 6. 10.1038/s43586-021-00092-5 37325194 PMC7614635

[B28] Shalapour S. Font-Burgada J. Di Caro G. Zhong Z. Sanchez-Lopez E. Dhar D. (2015). Immunosuppressive plasma cells impede T-cell-dependent immunogenic chemotherapy. Nature 521 (7550), 94–98. 10.1038/nature14395 25924065 PMC4501632

[B29] Shin A. E. Giancotti F. G. Rustgi A. K. (2023). Metastatic colorectal cancer: mechanisms and emerging therapeutics. Trends Pharmacol. Sci. 44 (4), 222–236. 10.1016/j.tips.2023.01.003 36828759 PMC10365888

[B30] Siegel R. L. Miller K. D. Fuchs H. E. Jemal A. (2022). Cancer statistics. CA Cancer J. Clin. 72 (1), 7–33. 10.3322/caac.21708 35020204

[B31] Siegel R. L. Giaquinto A. N. Jemal A. (2024). Cancer statistic. CA Cancer J. Clin. 74 (1), 12–49. 10.3322/caac.21820 38230766

[B32] Skrivankova V. W. Richmond R. C. Woolf B. A. R. Yarmolinsky J. Davies N. M. Swanson S. A. (2021). Strengthening the reporting of observational studies in epidemiology using mendelian randomization: the STROBE-MR statement. JAMA 326 (16), 1614–1621. 10.1001/jama.2021.18236 34698778

[B33] Smith L. C. Ramar M. Riley G. L. Mathias C. B. Lee J. Y. (2025). Steroid hormone regulation of immunometabolism and inflammation. Front. Immunol. 16, 1654034. 10.3389/fimmu.2025.1654034 41041336 PMC12483911

[B34] Snaebjornsson M. T. Janaki-Raman S. Schulze A. (2020). Greasing the wheels of the cancer machine: the role of lipid metabolism in cancer. Cell Metab. 31 (1), 62–76. 10.1016/j.cmet.2019.11.010 31813823

[B35] Sun J. Dong Q. Wei J. Gao Y. Yu Z. Hu X. (2025). ti-scMR: trajectory-inference-based dynamic single-cell Mendelian randomization identifies causal genes underlying phenotypic differences. Nar. Genom Bioinform 7 (3), lqaf082. 10.1093/nargab/lqaf082 40630931 PMC12231591

[B36] Võsa U. Claringbould A. Westra H. J. Bonder M. J. Deelen P. Zeng B. (2021). Large-scale cis- and trans-eQTL analyses identify thousands of genetic loci and polygenic scores that regulate blood gene expression. Nat. Genet. 53 (9), 1300–1310. 10.1038/s41588-021-00913-z 34475573 PMC8432599

[B37] Wan J. Liu H. Chu J. Zhang H. (2019). Functions and mechanisms of lysine crotonylation. J. Cell Mol. Med. 23 (11), 7163–7169. 10.1111/jcmm.14650 31475443 PMC6815811

[B38] Wang Z. A. Cole P. A. (2020). The chemical biology of reversible lysine post-translational modifications. Cell Chem. Biol. 27 (8), 953–969. 10.1016/j.chembiol.2020.07.002 32698016 PMC7487139

[B39] Wang F. Long J. Li L. Wu Z. X. Da T. T. Wang X. Q. (2023). Single-cell and spatial transcriptome analysis reveals the cellular heterogeneity of liver metastatic colorectal cancer. Sci. Adv. 9 (24), eadf5464. 10.1126/sciadv.adf5464 37327339 PMC10275599

[B40] Wang X. Qu Y. Li Z. Xia Q. (2025). Histone crotonylation in tumors. Mol. Clin. Oncol. 22 (5), 39. 10.3892/mco.2025.2834 40160299 PMC11948463

[B41] Wherry E. J. Kurachi M. (2015). Molecular and cellular insights into T cell exhaustion. Nat. Rev. Immunol. 15 (8), 486–499. 10.1038/nri3862 26205583 PMC4889009

[B42] Xie J.-y. Ju J. Zhou P. Chen H. Wang S. C. Wang K. (2024). The mechanisms, regulations, and functions of histone lysine crotonylation. Cell Death Discov. 10 (1), 66. 10.1038/s41420-024-01830-w 38331935 PMC10853258

[B43] Xiong X. Panchenko T. Yang S. Zhao S. Yan P. Zhang W. (2016). Selective recognition of histone crotonylation by double PHD fingers of MOZ and DPF2. Nat. Chem. Biol. 12 (12), 1111–1118. 10.1038/nchembio.2218 27775714 PMC5253430

[B44] Xu X. Peng Q. Jiang X. Tan S. Yang Y. Yang W. (2023). Metabolic reprogramming and epigenetic modifications in cancer: from the impacts and mechanisms to the treatment potential. Exp. & Mol. Med. 55 (7), 1357–1370. 10.1038/s12276-023-01020-1 37394582 PMC10394076

[B45] Yang P. Qin Y. Zeng L. He Y. Xie Y. Cheng X. (2023). Crotonylation and disease: current progress and future perspectives. Biomed. Pharmacother. 165, 115108. 10.1016/j.biopha.2023.115108 37392654

[B46] Yang K. Nong J. Xie H. Wan Z. Zhou X. Liu J. (2024a). DPF2 overexpression correlates with immune infiltration and dismal prognosis in hepatocellular carcinoma. J. Cancer 15 (14), 4668–4685. 10.7150/jca.97437 39006087 PMC11242344

[B47] Yang S. Fan X. Yu W. (2024b). Regulatory mechanism of protein crotonylation and its relationship with cancer. Cells 13 (21), 1812. 10.3390/cells13211812 39513918 PMC11545499

[B48] Yang Y. Chen X. Pan J. Ning H. Zhang Y. Bo Y. (2024c). Pan-cancer single-cell dissection reveals phenotypically distinct B cell subtypes. Cell 187 (17), 4790–4811.e22. 10.1016/j.cell.2024.06.038 39047727

[B49] Yazar S. Alquicira-Hernandez J. Wing K. Senabouth A. Gordon M. G. Andersen S. (2022). Single-cell eQTL mapping identifies cell type-specific genetic control of autoimmune disease. Science 376 (6589), eabf3041. 10.1126/science.abf3041 35389779

[B50] Yuan H. Wu X. Wu Q. Chatoff A. Megill E. Gao J. (2023). Lysine catabolism reprograms tumour immunity through histone crotonylation. Nature 617 (7962), 818–826. 10.1038/s41586-023-06061-0 37198486 PMC11089809

[B51] Yun Z. Guo Z. Li X. Shen Y. Nan M. Dong Q. (2023). Genetically predicted 486 blood metabolites in relation to risk of colorectal cancer: a Mendelian randomization study. Cancer Med. 12 (12), 13784–13799. 10.1002/cam4.6022 37132247 PMC10315807

[B52] Zhai G. Niu Z. Jiang Z. Zhao F. Wang S. Chen C. (2024). DPF2 reads histone lactylation to drive transcription and tumorigenesis. Proc. Natl. Acad. Sci. U. S. A. 121 (50), e2421496121. 10.1073/pnas.2421496121 39636855 PMC11648877

[B53] Zhang L. Li Z. Skrzypczynska K. M. Fang Q. Zhang W. O'Brien S. A. (2020). Single-Cell analyses inform mechanisms of Myeloid-targeted therapies in Colon cancer. Cell 181 (2), 442–459.e29. 10.1016/j.cell.2020.03.048 32302573

[B54] Zhao Y. Hao S. Wu W. Li Y. Hou K. Liu Y. (2022). Lysine crotonylation: an emerging player in DNA damage response. Biomolecules 12 (10), 1428. 10.3390/biom12101428 36291637 PMC9599786

[B55] Zheng J. Yang Q. Liu H. Zhao H. Wang S. Liu Y. (2025). Integrating single-cell transcriptome-wide mendelian randomization and differentially expressed gene analyses to prioritize dynamic immune-related drug targets for cancers. Adv. Sci. (Weinh) 12 (46), e07451. 10.1002/advs.202507451 41216857 PMC12697907

