# Correction: The crotonylation reader DPF2 promotes the development and progression of colon adenocarcinoma through cell-type-specific immune regulation and metabolic reprogramming

**DOI:** 10.3389/fphar.2026.1902042

**Published:** 2026-09-18

**Authors:** Jingzhi Wang, Jingyi Zhou, Lin Liu

**Affiliations:** 1 Department of Oncology, Zhongda Hospital, School of Medicine, Southeast University, Nanjing, Jiangsu, China; 2 Department of Radiotherapy Oncology, The First People’s Hospital of Yancheng, Yancheng No.1 People’s Hospital, Affiliated Hospital of Medical School, Nanjing University, Yancheng, Jiangsu, China

**Keywords:** colon adenocarcinoma, crotonylation, DPF2, metabolic reprogramming, tumor immune microenvironment

There was an inconsistency in Table 1 as published. To ensure consistency with the baseline staging definition used for the TNM stage variable in the same table, the values for the rows “lymph node metastasis” and “distant metastasis” have been updated according to the baseline clinicopathological staging records. The corrected Table 1 appears below. This correction does not alter the overall scientific conclusions of the article.

**TABLE 1:** Clinicopathological characteristics according to DPF2 expression in patients with colon adenocarcinoma.

Variable	Total (n = 60)	Low expression (n = 33)	High expression (n = 27)	P value
Age, years, median (IQR)	59.5 (54.0–70.0)	65.0 (58.0–70.0)	58.0 (52.0–64.0)	0.090
**Sex, n (%)**	​	​	​	1.000
Male	33 (55.0%)	18 (54.5%)	15 (55.6%)	​
Female	27 (45.0%)	15 (45.5%)	12 (44.4%)	​
**Differentiation, n (%)**	​	​	​	0.084
Well differentiated	17 (28.3%)	8 (24.2%)	9 (33.3%)	​
Moderately differentiated	34 (56.7%)	17 (51.5%)	17 (63.0%)	​
Poorly differentiated	9 (15.0%)	8 (24.2%)	1 (3.7%)	​
**TNM stage, n (%)**	​	​	​	**0.007**
Stage I	11 (18.3%)	9 (27.3%)	2 (7.4%)	​
Stage II	17 (28.3%)	13 (39.4%)	4 (14.8%)	​
Stage III	22 (36.7%)	8 (24.2%)	14 (51.9%)	​
Stage IV	10 (16.7%)	3 (9.1%)	7 (25.9%)	​
**Lymph node metastasis, n (%)**	​	​	​	**0.009**
No	32 (53.3%)	23 (69.7%)	9 (33.3%)	​
Yes	28 (46.7%)	10 (30.3%)	18 (66.7%)	​
**Distant metastasis, n (%)**	​	​	​	0.097
No	50 (83.3%)	30 (90.9%)	20 (74.1%)	​
Yes	10 (16.7%)	3 (9.1%)	7 (25.9%)	​
**Recurrence, n (%)**	​	​	​	0.397
No	42 (70.0%)	25 (75.8%)	17 (63.0%)	​
Yes	18 (30.0%)	8 (24.2%)	10 (37.0%)	​
**Vital status at last follow-up, n (%)**	​	​	​	**0.021**
Alive	28 (46.7%)	20 (60.6%)	8 (29.6%)	​
Dead	32 (53.3%)	13 (39.4%)	19 (70.4%)	​

Bold values indicate statistical significance (P < 0.05). Continuous variables are presented as median (interquartile range, IQR) and were compared using the Mann–Whitney U test. Categorical variables are presented as n (%), with percentages calculated within each column unless otherwise indicated, and were compared using Pearson’s chi-square test or Fisher’s exact test, as appropriate. TNM, tumor–node–metastasis; IQR, interquartile range.

The original article has been updated.

In the published article, in the Results section, Section 3.2, the following text was incorrectly given as: “high DPF2 expression was significantly associated with more advanced TNM stage (P = 0.007), as well as a higher likelihood of lymph node metastasis (P = 0.012) and distant metastasis (P = 0.026) (Table 1).” This is now corrected as follows:

“high DPF2 expression was significantly associated with more advanced TNM stage (P = 0.007) and lymph node metastasis (P = 0.009) (Table 1).”

There was a mistake in Figure 3D as published. The x‐axis labels of the Kaplan–Meier overall survival curve were incorrect. The corrected Figure 3 appears below. This correction does not affect the interpretation or the scientific conclusions of the article.

**FIGURE 3:**
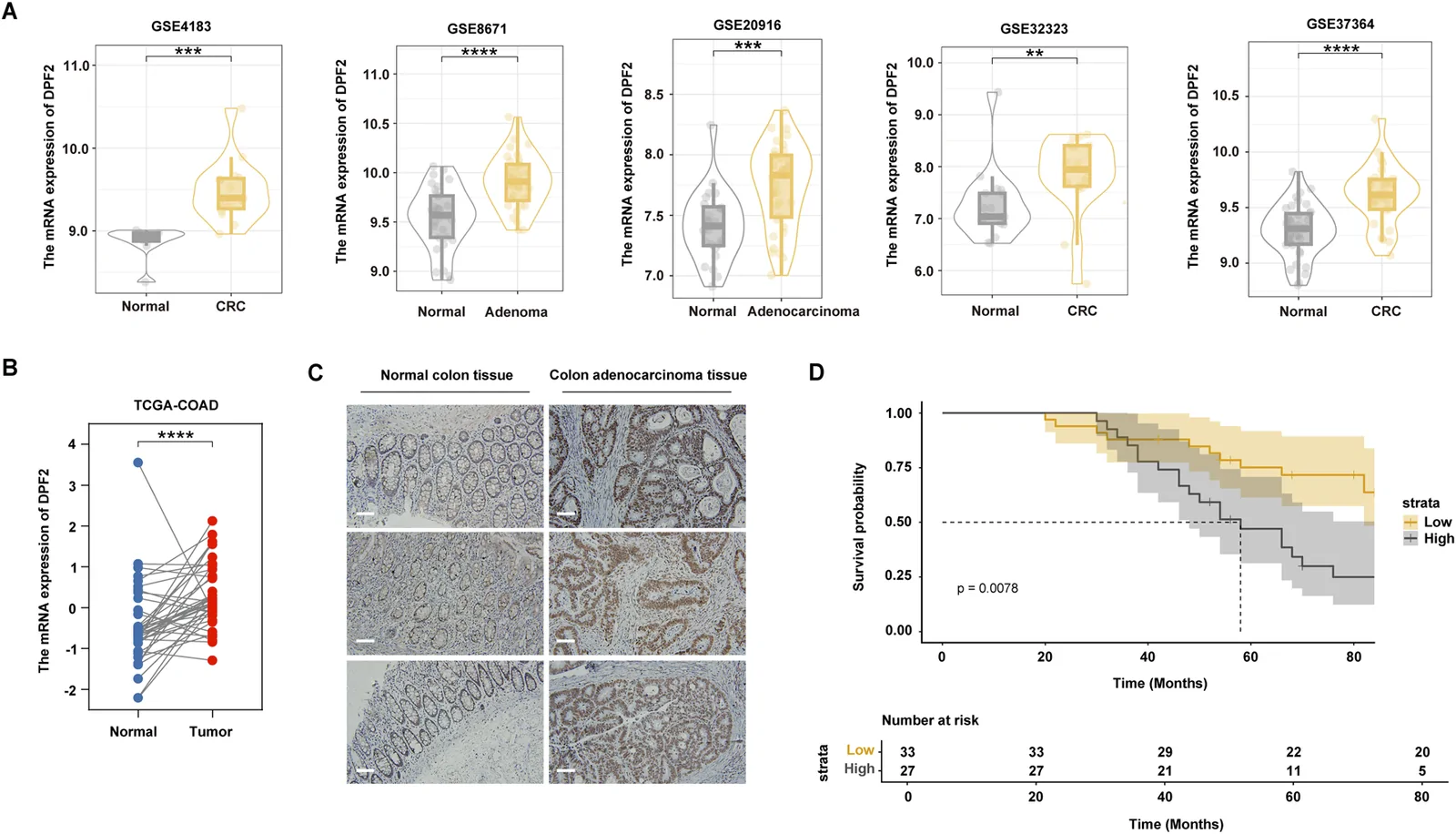
Validation of DPF2 expression and prognostic significance in colon adenocarcinoma. **(A)** Differential expression of DPF2 in normal colon tissues, adenoma tissues, and colon adenocarcinoma tissues across GEO cohorts. **(B)** Comparison of DPF2 expression between adjacent normal tissues and tumor tissues in the TCGA-COAD cohort. **(C)** Representative immunohistochemical staining images with scale bars (100 μm) showing DPF2 protein expression in normal colon tissues and colon adenocarcinoma tissues. **(D)** Kaplan–Meier overall survival analysis stratified by DPF2 expression level.

In the published Supplementary Material, the titles/designations of several Supplementary Tables did not fully correspond to their citations in the main text. The Supplementary Material has been updated only to correct the Supplementary Table titles/designations. No changes have been made to the underlying supplementary data, numerical results, statistical analyses, interpretation, or conclusions.

