# Supplementary figures and images for: The crotonylation reader DPF2 promotes the development and progression of colon adenocarcinoma through cell-type-specific immune regulation and metabolic reprogramming
Source: Front Pharmacol. 2026 May 18;17:1835967. doi: 10.3389/fphar.2026.1835967 (PMC13223100; doi:10.3389/fphar.2026.1835967)

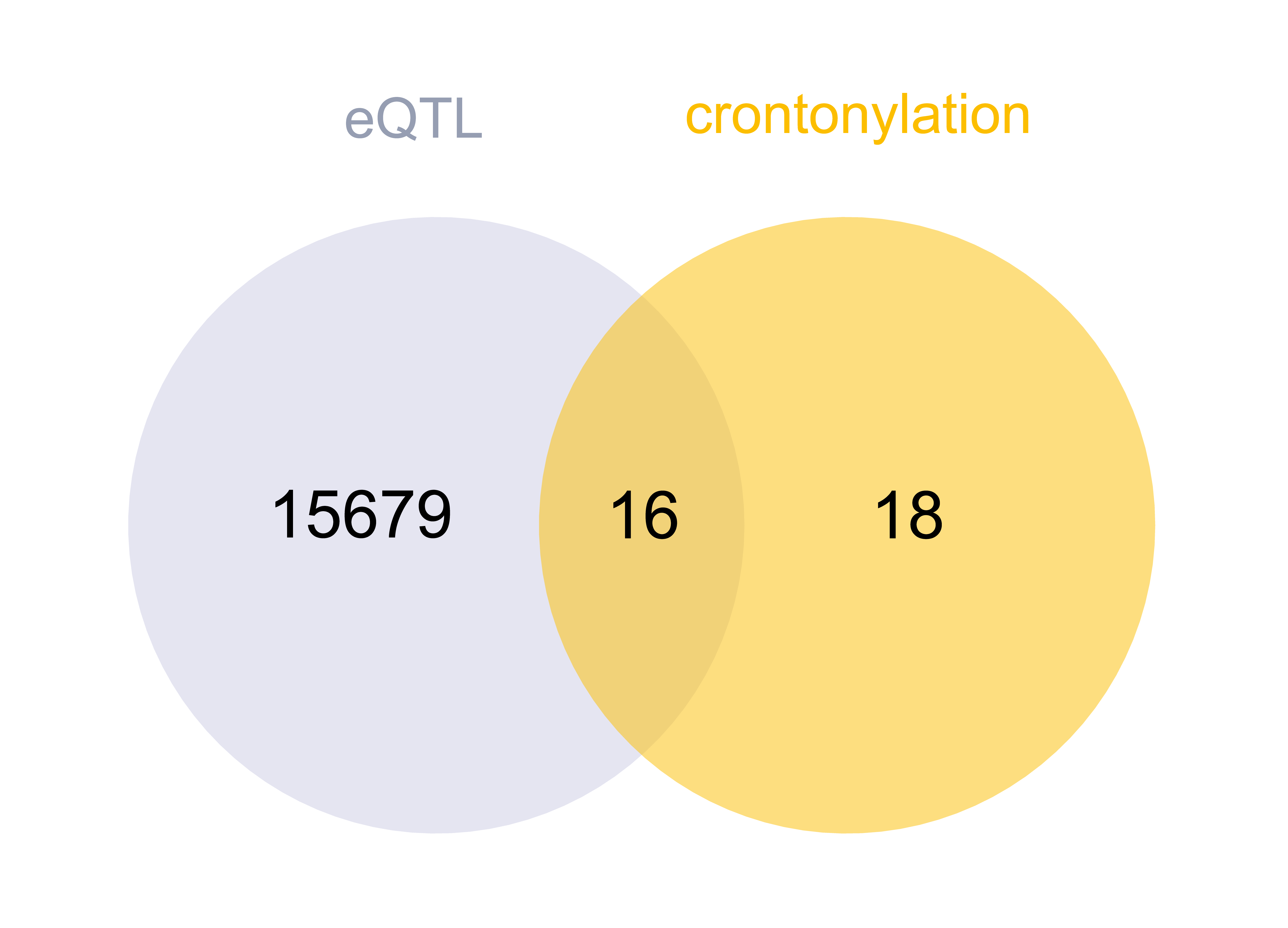

Supplement: Supplementary file 1 [file Image1.tif]
